# Interaction networks of SIM-binding groove mutants reveal alternate modes of SUMO binding and profound impact on SUMO conjugation

**DOI:** 10.1126/sciadv.adp2643

**Published:** 2025-05-14

**Authors:** Laura A. Claessens, Matty Verlaan–de Vries, Nienke Broekman, Martijn S. Luijsterburg, Alfred C. O. Vertegaal

**Affiliations:** ^1^ Department of Cell and Chemical Biology, Leiden University Medical Center, Leiden, Netherlands.; ^2^ Department of Human Genetics, Leiden University Medical Center, Leiden, Netherlands.

## Abstract

The best-characterized mode of noncovalent SUMO interaction involves binding of a SUMO-interaction motif (SIM) to a conserved binding groove in SUMO. Our knowledge on other types of SUMO interactions is still limited. Using SIM-binding groove SUMO2/3 mutants and mass spectrometry, we identified proteins that bind to SUMO in an alternate manner. Domain-enrichment analysis characterized a group of WD40 repeat domain–containing proteins as SIM-independent SUMO interactors, and we validated direct binding of SEC13 and SEH1L to SUMO in vitro*.* Using AlphaFold-3 modeling and in vitro mutational analysis, we identified residues in the WD40 domain of SEC13 and SUMO2/3’s C terminus involved in the interaction. Furthermore, SIM-binding groove mutants failed to interact with SUMO E3 ligases belonging to the PIAS family, RANBP2, ZNF451, and TOPORS, leading to loss of covalent conjugation to most of SUMO target proteins. Together, our dataset serves as a unique resource and offers valuable insights on the intricacies of the SUMO interactome and SUMO targets.

## Teaser

Many proteins, including a group of WD40 proteins, bind to SUMO independently of the classical SUMO-SIM mechanism.

## INTRODUCTION

Posttranslational modification of proteins by small ubiquitin-like modifier (SUMO) proteins is essential for many cellular processes, including transcription, genome stability, and cell cycle progression (*1*). In mammalian cells, SUMO1, SUMO2, and SUMO3 are the major SUMO paralogs. SUMO2 and SUMO3, collectively termed SUMO2/3, are almost identical and share ~50% sequence identity with SUMO1. Covalent attachment of SUMO to lysine residues in target proteins is orchestrated in an adenosine 5′-triphosphate (ATP)–dependent manner by an enzymatic cascade involving the SUMO-activating enzyme SAE1/UBA2, the SUMO E2-conjugating enzyme UBC9, and SUMO E3 ligases. SUMOylation can occur on a single lysine residue (monoSUMOylation) or multiple lysine residues (multi-monoSUMOylation) in a target protein. SUMO2 and SUMO3 also form polymeric SUMO chains (polySUMOylation), mainly through modification of their internal K11 residues (*2*, *3*). SUMOylation is a dynamic and reversible process. Through the action of sentrin-specific SUMO proteases (SENPs), SUMO is removed from target proteins and recycled to maintain homeostasis (*4*).

Besides covalent modification, SUMO also affects protein function through noncovalent interactions (*5*). Three types of noncovalent SUMO interactions are distinguished. The typical class I SUMO interaction is between a SUMO-interaction motif (SIM) in the interacting protein and a conserved hydrophobic groove in SUMO formed by the second β sheet and the α helix (the SIM-binding groove) (*6*, *7*). SIMs consist of a short stretch of hydrophobic residues, surrounded by an acidic region (*8*). SUMO-SIM interactions can be modulated by acetylation of specific lysines in SUMO or phosphorylation near the hydrophobic core of SIMs (*9*–*14*). SUMO-SIM interactions are the best-characterized and most predominant mode of SUMO interaction. The class II SUMO interaction involves a surface on SUMO opposite of the SIM-binding groove. UBC9, RNF111, and DPP9 were the first proteins identified to bind to SUMO in this manner, although they do not all bind to an identical but rather a similar surface. SUMO binding of UBC9 is paralog-independent, while DPP9 and RNF111 show selectivity for SUMO1 (*15*–*18*). Recently, a cross-linking approach to specifically capture class II SUMO1 interactors identified 192 proteins that bound preferentially through class II interaction, including the previously validated UBC9 and DPP9 (*19*). Class III SUMO interaction involves binding to a distinct region of SUMO1 via a ZZ-zinc finger motif (*15*). Two proteins, HERC2 and CBP, bind to SUMO through this mode of interaction (*20*, *21*). It is unknown whether more proteins interact with SUMO through class II and III interactions or through yet to be discovered modes of interaction.

Multiple independent mass spectrometry (MS) screens have been performed in the last decade to gain more insight into the SUMO interactome (*19*, *22*–*26*). These studies have mainly focused on global identification of interactors binding to different SUMO isoforms and topologies rather than distinguishing modes of interaction. Using different approaches, such as microarray and pull-down strategies, hundreds of SUMO interactors have been identified (*22*–*25*). In another study, a covalent cross-linking approach was used to also capture low-affinity interactors that might be lost during abovementioned approaches (*26*). SUMO interactors identified in these screens include proteins involved in a variety of cellular processes, such as transcription, cell cycle, and DNA repair. A different mode of SUMO interaction was first discovered through zinc finger proteins ZNF198 (ZMYM2) and ZNF261 (ZMYM3), where the MYM-type zinc fingers are necessary and sufficient for SUMO binding (*27*). This is an example of SUMO binding that occurs independently of a conventional SIM, although these zinc fingers do bind to the SIM-binding groove of SUMO. Furthermore, the DNA repair protein XRCC4 was recently reported to interact with the SIM-binding groove of SUMO through two binding surfaces that are similar but distinct from conventional SIMs in that they consist of a short hydrophobic stretch with a positive charge at the core (*22*). Because the class I SUMO-SIM interaction appears to be the most abundant mode of interaction, most of datasets, so far, will automatically consist predominantly of proteins using this mode of SUMO interaction, making it difficult to select for proteins that might bind in an alternate manner. The recent identification of 192 proteins binding preferentially to SUMO1 through class II interaction rather than class I interaction, supports the notion that we are still limited in our knowledge on noncovalent SUMO interactions beyond the classical SUMO-SIM interaction (*19*).

Here, we sought out to specifically screen for SUMO interactors that do not bind to the conserved SIM-binding groove of SUMO but interact through other regions. Using SIM-binding groove SUMO2/3 mutants, we performed a pull-down screen to capture proteins capable of binding these SUMOs and, thereby, specifically enrich for proteins that bind to SUMO in an alternate manner. By comparing the SUMO2/3 mutant interactomes with the SUMO2/3 wild-type (WT) interactome, we identified proteins exclusively binding to SUMO2/3 WT and proteins capable of binding both. Unexpectedly, we found that many proteins were able to bind both SUMO2/3 WT and the mutants, suggesting that the prevalence and relevance of SIM-independent modes of SUMO interaction are now underestimated. We confirmed UBC9 as one of these interactors and also identified the SUMO E1 enzyme SAE1/UBA2 and the SUMO proteases SENP1-2 and SENP5. Other SUMO machinery components do seem to require the SIM-binding groove for binding, especially SUMO E3 ligases. Furthermore, we identified a group of WD40 domain–containing proteins as a class of SIM-independent SUMO interactors and validated direct binding of SEC13 and SEH1L to SUMO in vitro*.* By using AlphaFold-3–based structural modeling for the SEC13 interaction with SUMO, we identified and experimentally validated residues in the WD40 domain of SEC13 and SUMO2/3’s C terminus that mediate this interaction. Additionally, we extended our understanding of the SUMO interactome to also include covalent SUMO modification in the context of the SIM-binding groove. Together, our dataset represents a unique resource and offers valuable insights on the intricacies of the SUMO interactome.

## RESULTS

### A noncovalent SUMO-binder screen with SIM-binding groove SUMO2/3 mutants

**Fig. 1.**
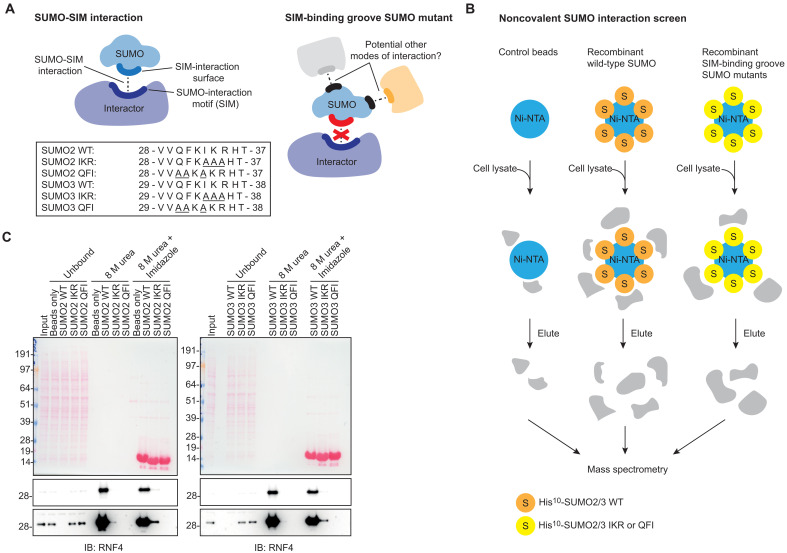
A noncovalent SUMO interactomics screen to identify SIM-independent SUMO binders. (**A**) Cartoon illustrating the typical class I SUMO-SIM interaction and the concept of a SIM-binding groove SUMO mutant to capture alternate modes of interaction. The box depicts the SUMO variants used in the screen and corresponding sequences around the mutation site. (**B**) Cartoon illustrating the experimental setup of the screen. Empty nickel–nitrilotriacetic acid (Ni-NTA) beads and beads coated with either SUMO2/3 wild-type (WT), QFI, or IKR were incubated with HeLa lysates, and noncovalent SUMO interactors were purified and identified by mass spectrometry (MS). (**C**) Immunoblotting (IB) of a representative replicate of the screen, *n* = 4 (top: short exposure; bottom: long exposure). RNF4 was used as a control for binding to SUMO2/3 WT, but not the SIM-binding groove mutants. (C) Duplicated in the replicate 1 panel in fig. S1B.

To identify potential alternate modes of noncovalent SUMO interaction, 10xHis-tagged versions of SUMO2 and SUMO3 WT and SIM-binding groove mutants were recombinantly produced in *Escherichia coli* and purified (Fig. 1A and fig. S1A). We included two SIM-binding groove SUMO mutants in which the hydrophobic surface of SUMO that mediates the SUMO-SIM interaction was mutated, I33A-K34A-R35A (SUMO2 IKR), Q30A-F31A-I33A (SUMO2 QFI), I34A-K35A-R36A (SUMO3 IKR), and Q31A-F32A-I34A (SUMO3 QFI). Next, the His^10^-tagged SUMO variants were coupled to nickel–nitrilotriacetic acid (Ni-NTA) beads and incubated with whole-cell HeLa lysates. Uncoated beads were used as negative control. Proteins binding to the SUMO proteins were eluted, trypsin digested, and analyzed by MS (Fig. 1B). Proteins bound to SUMO were first eluted from the beads with 8 M urea buffer, allowing identification of noncovalent interactors. A second elution with 8 M urea buffer and imidazole was performed to elute any remaining interactors together with the SUMO proteins from the beads. Four biological replicates were performed. Immunoblotting with RNF4 confirmed efficient binding to WT SUMO2 and SUMO3 and abrogated binding to SUMO2/3 IKR and QFI (Fig. 1C and fig. S1B). Proteins binding to the SUMO2 and SUMO3 WT and mutants were identified and quantified by MS. After removing common contaminants and non-consistently identified proteins, further analysis was performed for 1434 and 1321 protein groups for SUMO2 (table S1) and SUMO3 (table S2), respectively. Heatmap analysis (fig. S2A) and principal components analysis (PCA) clustering (fig. S2B) of the identified proteins showed clustering of biological replicates by experimental condition, indicating high reproducibility.

### Overlap between SUMO2 and SUMO3 interactors

**Fig. 2.**
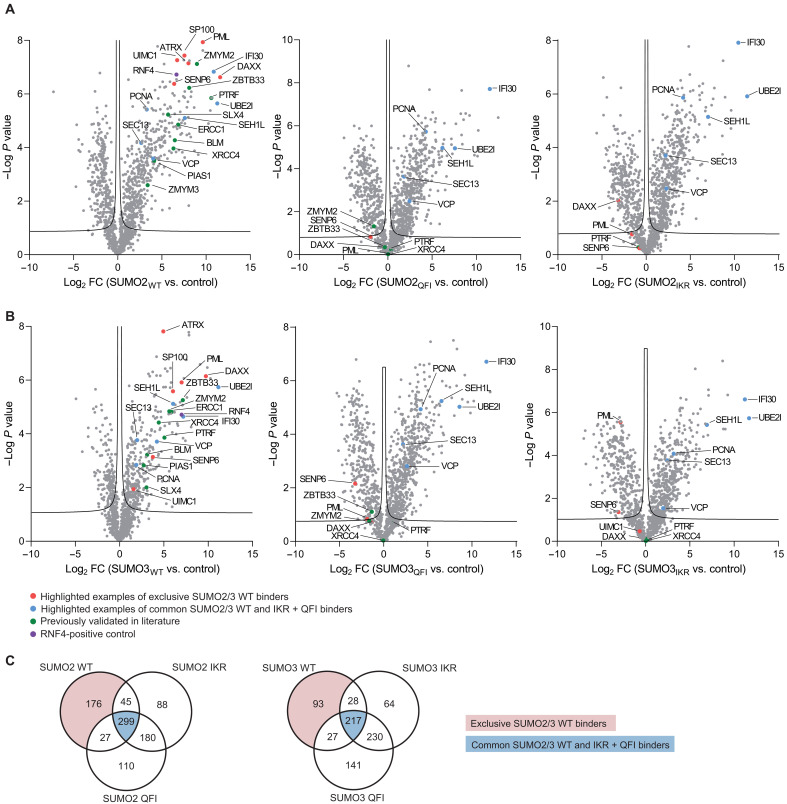
Identification of exclusive SUMO2/3 WT and SIM-independent SUMO2/3 binders. Volcano plots depicting the interactors for (**A**) SUMO2 WT, QFI, and IKR and (**B**) SUMO3 WT, QFI, and IKR compared to control beads. Proteins are represented by dots. Red dots represent highlighted examples of exclusive SUMO2/3 WT binders; green dots represent proteins previously validated in literature to bind SUMO in a SIM-dependent manner; blue dots represent highlighted examples of common SUMO2/3 WT and IKR/QFI binders; purple dot represents positive control RNF4. Significance was determined with a Student’s *t* test, false discovery rate (FDR) = 0.05 and *s0* = 0.1, *n* = 4 independent experiments. FC, fold change. (**C**) Venn diagrams for the identified SUMO2 (left) and SUMO3 interactors (right). The exclusive SUMO2/3 WT binders are highlighted in red, and the common SUMO2/3 WT/QFI/IKR binders are highlighted in blue.

Mature SUMO2 and SUMO3 differ from each other in their N-terminal tail regions. Previous SUMO interactome screens have looked at interactors of SUMO1 versus SUMO2/3 versus polymeric SUMO2/3 chains, but not at potential differences in interactors between SUMO2 and SUMO3 within the same screen (*19*, *23*–*25*). Compared to the uncoated bead-control sample, we identified 547 significant SUMO2 WT, 612 significant SUMO2 IKR, and 616 significant SUMO2 QFI interactors [*t* test, false discovery rate (FDR) = 0.05, log_2_ fold change > 0] (Fig. 2A). Compared to the uncoated bead-control sample, we identified 365 significant SUMO3 WT, 539 SUMO3 IKR, and 615 SUMO3 QFI interactors (*t* test, FDR = 0.05, log_2_ fold change > 0) (Fig. 2B). Comparing the SUMO3 interactome with the SUMO2 interactome, we identified less SUMO3 WT interactors than SUMO2 WT interactors (*n* = 365 versus *n* = 547, respectively). Likewise, when overlapping the SUMO2 and SUMO3 WT binders, we identified 320 overlapping binders and more unique SUMO2 binders (*n* = 227) than unique SUMO3 binders (*n* = 45) (fig. S3A). We performed STRING analysis and MCODE clustering on the unique SUMO3 binders (fig. S3B). To strengthen our MS data, we confirmed exclusive or preferential binding of 53BP1, RAD18, ZNF451, RMI1, and RMI2 to SUMO2 compared to SUMO3 by immunoblotting, while RNF4 showed equal binding (fig. S3C). The unique SUMO2 and SUMO3 binders potentially represent isoform-specific interactors, perhaps partly facilitated by the differences in their N-terminal tail regions. Because the SIM-binding groove and surrounding region of SUMO are identical between SUMO2 and SUMO3, the overlapping proteins are of main interest when delineating the dependency of the SUMO interactome in this context.

### SUMO-SIM interaction–dependent SUMO2/3 binders

A total of 176 SUMO2 interactors bound exclusively to SUMO2 WT and 371 to both SUMO2 WT and at least one of the SIM-binding groove mutants (IKR and/or QFI); 299 proteins bound to all three SUMO2 proteins (Fig. 2C). Ninety-three SUMO3 interactors bound exclusively to SUMO3 WT and 217 to both SUMO3 WT and both the SIM-binding groove mutants (IKR and QFI) (Fig. 2C).

**Fig. 3.**
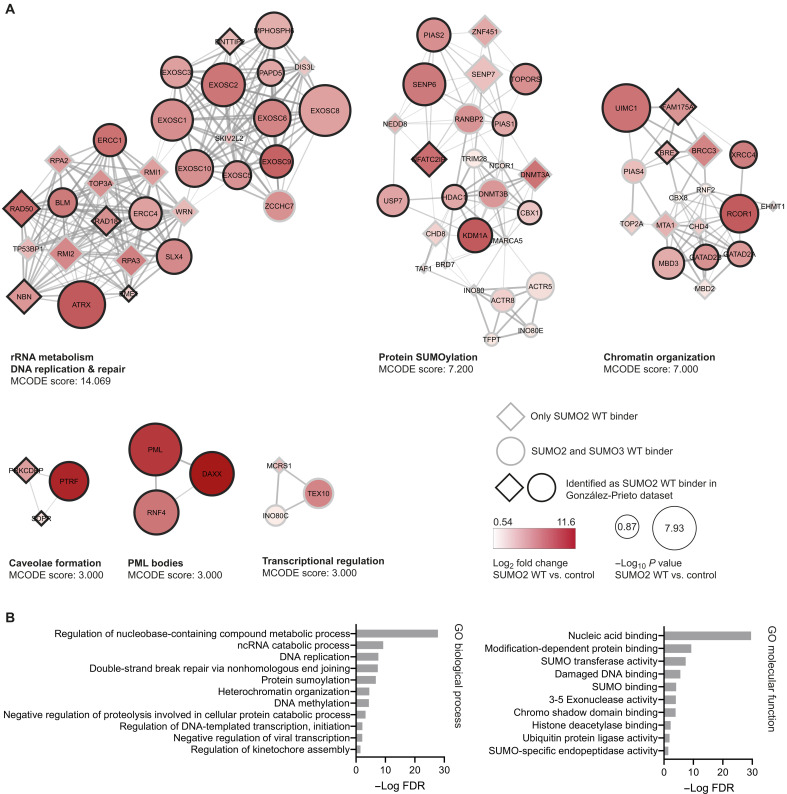
Analysis of exclusive SUMO2/3 WT binders. (**A**) STRING network and MCODE clustering analysis of the exclusive SUMO2/3 WT binders. MCODE scores and associated biological processes are included for each cluster. Circles represent proteins identified as both SUMO2 and SUMO3 binder; diamonds represent proteins only identified as SUMO2 binder. Black border indicates proteins that we also identified as SUMO2 WT binders in a previous interactomics screen. Node color and size indicate the log_2_ fold change and −log *P* value, respectively, of the enriched proteins compared to the control beads. rRNA, ribosomal RNA. (**B**) Gene Ontology (GO) enrichment analysis of the exclusive SUMO2/3 WT binders for biological process and molecular function. Bars indicate the −log FDR of the enrichment compared to the reference dataset. ncRNA, noncoding RNA.

The 176 proteins binding exclusively to SUMO2 WT and 93 proteins binding exclusively to SUMO3 WT and not SUMO2/3 QFI and IKR represent proteins that interact with SUMO through the SIM-binding groove of SUMO. They likely also represent proteins that interact with this surface through a consensus SIM, although tandem repeats of zinc fingers can also bind to this surface on SUMO (*27*). Because most of the SUMO3 WT interactomes overlapped with the SUMO2 WT interactomes as described above, we performed the follow-up analyses described below on the SUMO2 interactome, indicating which interactors were additionally identified in the SUMO3 interactome. We performed STRING analysis (fig. S4), MCODE clustering (Fig. 3A), and Gene Ontology (GO) enrichment (Fig. 3B) on these proteins and identified protein clusters corresponding to RNA metabolism, DNA repair, protein SUMOylation, and regulation of transcription.

In line with the immunoblotting validation of the screen setup, MS results identified RNF4 as significant SUMO2/3 WT interactor, but not a SUMO2/3 IKR and QFI interactor. ZMYM2, ZMYM3, BLM, PIAS1, PTRF, and ZBTB33 were previously shown to bind to SUMO through the SIM-binding groove (*27*, *28*), and, accordingly, we identified these proteins as SUMO2/3 WT interactors in our screen, but not as SUMO2/3 QFI and IKR interactors. We have previously shown that mutating the SIM in SLX4, ERCC1, and XRCC4 abrogates their interaction with SUMO, indicating that SLX4, ERCC1, and XRCC4 binding to SUMO is mediated through interaction of their SIM with the SIM-binding groove of SUMO (*25*, *29*, *30*). Consistently, SLX4, ERCC1, and XRCC4 were identified in our screen as exclusively interacting with SUMO2/3 WT and not with SUMO2/3 QFI and IKR.

### SUMO-SIM interaction–independent SUMO2/3 binders

**Fig. 4.**
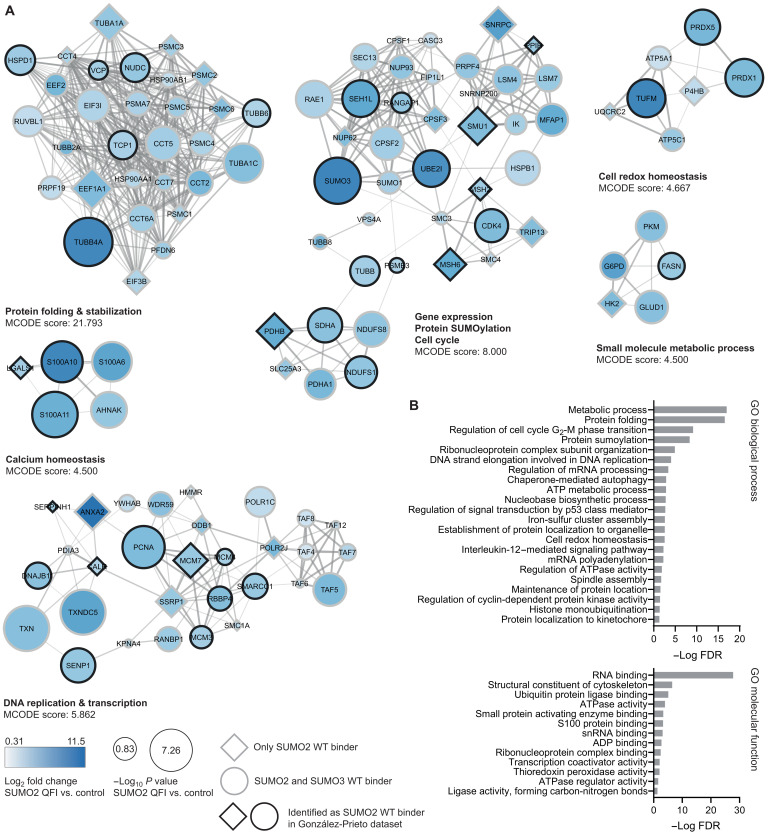
Analysis of the common SUMO2/3 WT and SIM-binding groove mutant binders. (**A**) STRING network and MCODE clustering analysis of the common SUMO2/3 WT/QFI/IKR binders. MCODE scores and associated biological processes are included for each cluster. The top six MCODE clusters are shown. Circles represent proteins identified as both SUMO2 and SUMO3 binder; diamonds represent proteins only identified as SUMO2 binder. Black border indicates proteins that we also identified as SUMO2 WT binders in a previous interactomics screen. Node color and size indicate the log_2_ fold change and −log *P* value, respectively, of the enriched proteins compared to the control beads. (**B**) GO enrichment analysis of the common SUMO2/3 binders for biological process and molecular function. Bars indicate the −log FDR of the enrichment compared to the reference dataset. ATP, adenosine 5′-triphosphate; ADP, adenosine 5′-diphosphate.

Of the 547 significant SUMO2 WT binders, 299 proteins were also identified as SUMO2 QFI and SUMO2 IKR binders, and, of the 365 significant SUMO3 WT binders, 217 proteins were also identified as SUMO3 QFI and IKR binders, indicating that these proteins do not bind to the SIM-binding groove of SUMO but potentially to another region of SUMO and not through conventional SUMO-SIM interactions. We performed STRING analysis (fig. S5), MCODE clustering (Fig. 4A and fig. S6), and GO enrichment (Fig. 4B) on these proteins and identified protein clusters corresponding to protein folding, cell cycle, DNA replication, and protein SUMOylation. Using GPS-SUMO, we found that 101 of the 176 (57%) exclusive SUMO2 WT binders have ≥1 high-probability SIM versus 114 of the 299 (38%) common SUMO2 WT/QFI/IKR binders, illustrating the ability of our screen to differentially enrich for SIM-dependent and SIM-independent SUMO interactors.

Although these proteins were identified in our screen as interactors of both the WT and SIM-binding groove SUMOs when compared to control beads, they might still bind preferentially to SUMO WT. To address this, we compared protein enrichment for SUMO2/3 WT with SUMO2/3 IKR and SUMO2/3 WT with SUMO2/3 QFI. Of the 299 common SUMO2 binders, only 23 proteins were significantly enriched for SUMO2 WT compared to SUMO2 IKR and QFI (fig. S7A). Of the 217 common SUMO3 binders, only 10 proteins were significantly enriched for SUMO3 WT compared to SUMO3 IKR and QFI, of which 8 overlapped with SUMO2 (fig. S7A). This indicates that most of the common WT and IKR/QFI binders showed similar enrichment levels, with most of the binders enriched in SUMO WT compared to the SIM-binding groove mutants being a consequence of exclusive binding to SUMO WT and not preferential binding.

Recently, others attempted to identify class II SUMO1 binders using a photocrosslinking approach (*19*). Class II SUMO binders use a surface opposite to the class I SUMO-SIM binding groove. DPP9 was shown to bind this surface on SUMO1, but not SUMO2/3, while UBC9 interaction with this surface was independent of SUMO paralogs (*16*–*18*). Comparing our SUMO2/3 WT/QFI/IKR binders with the SUMO1 backside binders identified in that screen, we found 23 overlapping proteins, including UBC9 (fig. S7B). Thus, these could be proteins that do not interact with SUMO2/3 via class I SUMO binding through SUMO-SIM interactions but rather interact with SUMO via class II binding, in a paralog-independent manner.

### Noncovalent SUMO binding of the Ub/Ubl machinery

**Fig. 5.**
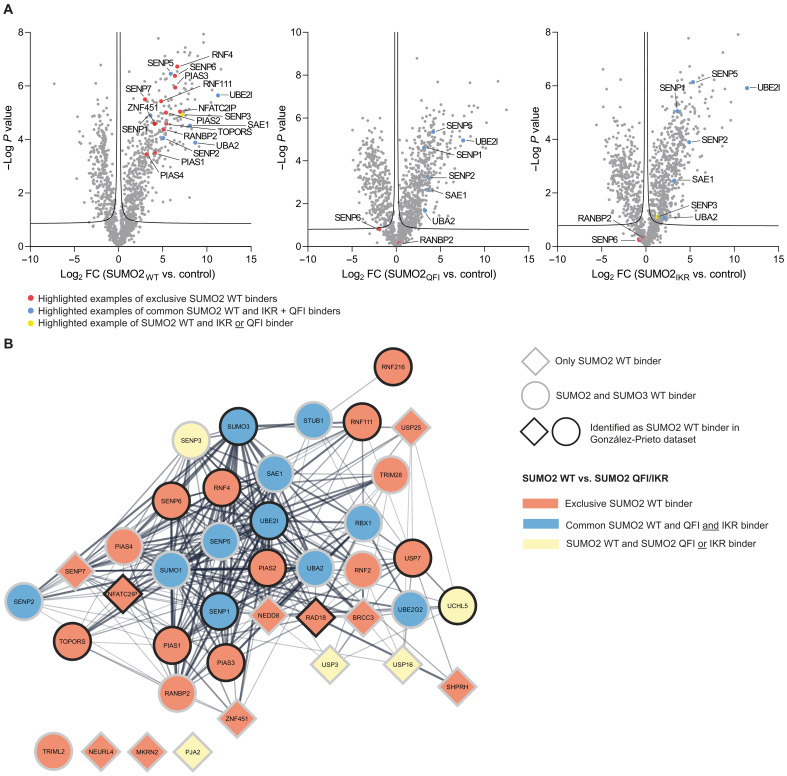
Noncovalent SUMO binding of the Ub/Ubl machinery. (**A**) Volcano plots from Fig. 2A depicting the interactors for SUMO2 WT, QFI, and IKR compared to control beads, respectively. Proteins are represented by dots. Red dots represent highlighted examples of the Ub/Ubl machinery exclusively binding to SUMO2 WT; blue dots represent highlighted examples of the Ub/Ubl machinery binding to SUMO2 WT/QFI/IKR; yellow dots represent highlighted examples of the Ub/Ubl machinery binding to SUMO2 WT and IKR or QFI. (**B**) STRING network of the identified Ub/Ubl proteins. Circles represent proteins identified as both SUMO2 and SUMO3 binder; diamonds represent proteins only identified as SUMO2 binder. Black border indicates proteins that we also identified as SUMO2 WT binders in a previous interactomics screen. Node color indicates whether proteins were identified as exclusive SUMO2/3 WT binder (red), SUMO2/3 WT/QFI/IKR binder (blue), or SUMO2/3 and IKR or QFI binder (yellow).

Further zooming in onto the protein SUMOylation cluster and expanding this to the ubiquitin (Ub)/ubiquitin-like (Ubl) machinery, we identified multiple components of these enzymatic machineries to interact exclusively with SUMO2 WT (Fig. 5, A and B). SUMO E3 ligases PIAS1-4, RANBP2, TOPORS, and ZNF451 exclusively interacted with SUMO2 WT, as well as SUMO-chain deconjugating proteases SENP6 and SENP7. The STUbLs RNF4 and RNF111 exclusively interacted with SUMO2 WT. NFATC2IP, described to down-regulate SUMO chain formation by UBC9, and NEDDylation protein NEDD8, exclusively interacted with SUMO2 WT. Some components were identified as both SUMO2 WT and SUMO2 QFI/IKR interactors: SENP1, SENP2, SENP3 (N.B.: only an IKR interactor, not a QFI interactor), and SENP5; the SUMO-activating enzyme subunits SAE1 and UBA2; and SUMO-conjugating enzyme UBC9. However, SENP5, SAE1, and UBA2 did show a preference for interacting with SUMO2 WT compared to both QFI and IKR. SENP3 preferentially bound SUMO2 WT over SUMO2 IKR.

### Searching for alternate SUMO interaction domains

**Fig. 6.**
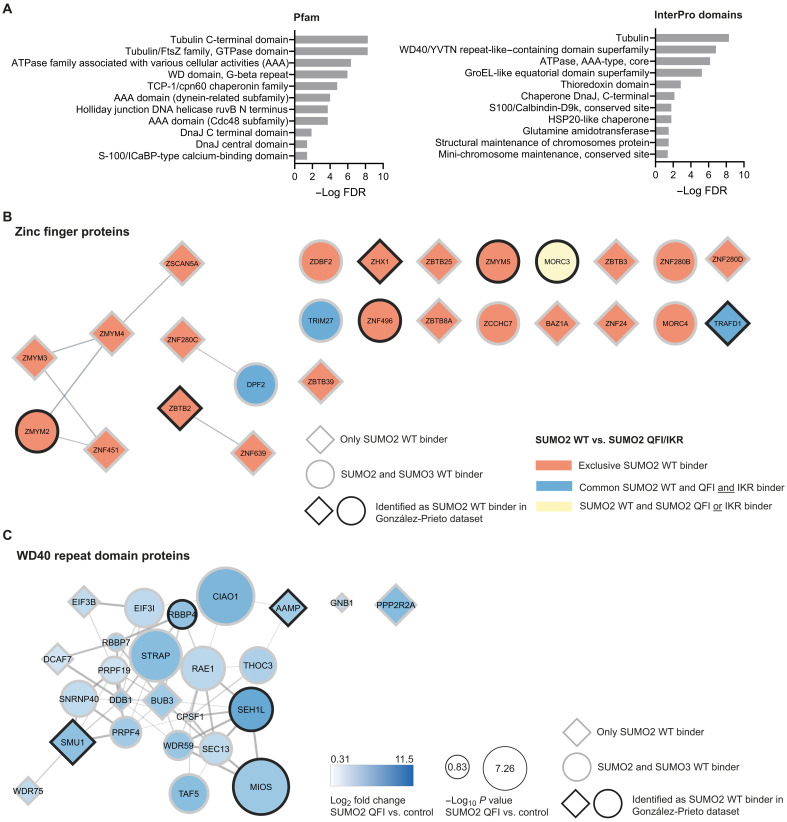
Domain enrichment analysis of SIM-dependent and SIM-independent SUMO2/3 binders. (**A**) Domain enrichment analysis using Pfam (left) and InterPro databases (right). Bars indicate the −log FDR of the enrichment compared to the reference dataset. GTPase, guanosine triphosphatase. (**B**) STRING network of zinc finger proteins binding to SUMO2/3. Circles represent proteins identified as both SUMO2 and SUMO3 binder; diamonds represent proteins only identified as SUMO2 binder. Black border indicates proteins that we also identified as SUMO2 WT binders in a previous interactomics screen. Node color indicates whether proteins were identified as exclusive SUMO2/3 WT binder (red), SUMO2/3 WT/QFI/IKR binder (blue), or SUMO2/3 and IKR or QFI binder (yellow). (**C**) STRING network of WD40 repeat domain proteins. Circles represent proteins identified as both SUMO2 and SUMO3 binder; diamonds represent proteins only identified as SUMO2 binder. Black border indicates proteins that we also identified as SUMO2 WT binders in a previous interactomics screen. Node color and size indicate the log_2_ fold change and −log *P* value, respectively, of the enriched proteins compared to the control beads.

Enrichment analysis using Pfam and InterPro databases identified tubulin, adenosine triphosphatase (ATPase), WD40 repeat, TCP-1/cpn60 chaperonin family, ATPases associated with diverse cellular activities (AAA), DNA J, and S-100 ICaBP–type calcium binding domain as significantly enriched domains among the common SUMO2 WT and IKR/QFI binders (Fig. 6A). In line with a previous report describing the binding of MYM-type zinc fingers to the SIM-binding groove of SUMO (*27*), zinc finger protein domains were only significantly enriched among exclusive SUMO2 WT binders. In addition to ZMYM2 and ZMYM3, which were previously shown to bind the SIM-binding groove via their MYM-type zinc fingers, we identified ZMYM4 and ZMYM5 as exclusive SUMO2 WT binders, as well as other zinc finger containing proteins (Fig. 6B).

**Fig. 7.**
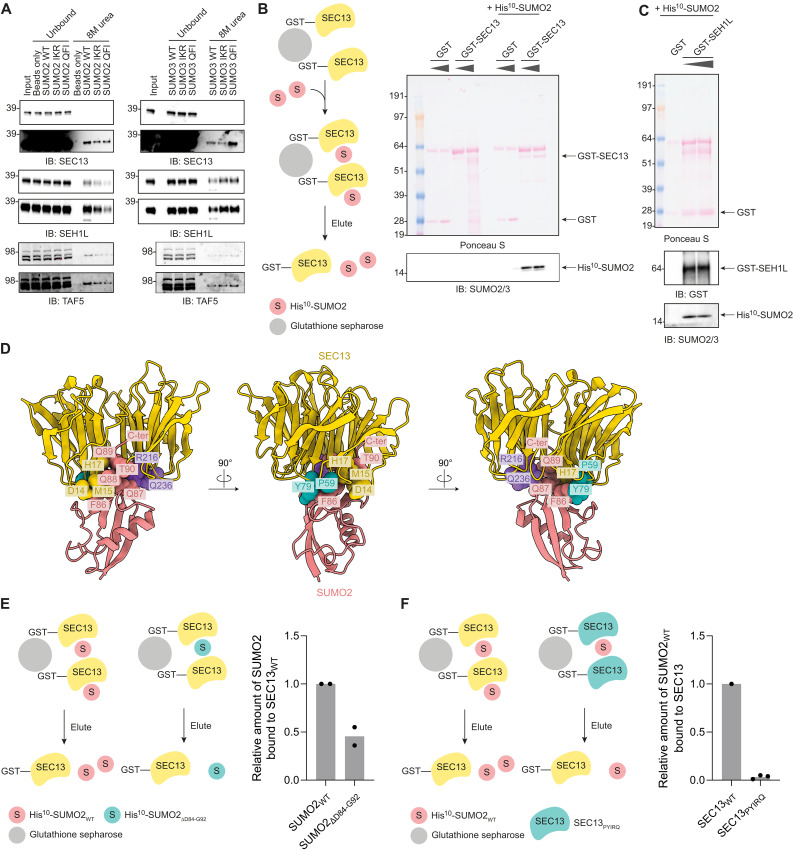
WD40 repeat domain proteins bind directly to SUMO2/3 in a SIM-independent manner. (**A**) Immunoblot verification of a representative replicate of the screen to confirm binding of the WD40 repeat domain proteins SEC13, SEH1L, and TAF5 to both SUMO2/3 WT and the SIM-binding groove mutants. A short (top) and a long (bottom) exposure are shown. (**B**) Cartoon depicting an in vitro glutathione *S*-transferase (GST)–pull-down assay with recombinant GST-SEC13. Elutions were analyzed by immunoblotting. GST only was used as a control for a specific binding. GST and GST-SEC13 were visualized by Ponceau S staining. Immunoblotting for SUMO2/3 confirmed binding of His^10^-SUMO2 to GST-SEC13. *n* = 3 independent experiments. (**C**) Similar as in (B), but for GST-SEH1L. GST and GST-SEH1L were visualized by Ponceau S staining and immunoblotting for GST. Immunoblotting for SUMO2/3 confirmed binding of His^10^-SUMO2 to GST-SEH1L. *n* = 3 independent experiments. (**D**) AlphaFold-3 prediction modeling of the SUMO-SEC13 interaction. Key residues involved in the interaction are highlighted. (**E**) Cartoon depicting an in vitro GST–pull-down assay with recombinant GST-SEC13 and His^10^-SUMO2 WT versus ∆D84-G92. Elutions were analyzed by SEC13 and SUMO2/3 immunoblotting. The amount of bound SUMO2 was quantified with SUMO2 WT set to 1.0 and plotted in a bar graph. Bar height indicates the mean. Dots represent replicate data. *n* = 2 independent experiments. (**F**) Cartoon depicting an in vitro GST–pull-down assay with recombinant GST-SEC13 WT versus P59A-Y79A-I151A-R216A-Q236A mutant and His^10^-SUMO2 WT. Elutions were analyzed by SEC13 and SUMO2/3 immunoblotting. The amount of bound SUMO2 was normalized to the amount of SEC13 present on the beads, quantified with SEC13 WT set to 1.0, and plotted in a bar graph. Bar height indicates the mean. Dots represent replicate data. *n* = 3 independent experiments.

The WD40 repeat domain was present in 25 of the common SUMO2 WT and IKR/QFI binders (Fig. 6C). This domain has been described previously as a ubiquitin binding domain in bacteria and yeast (*31*). We therefore explored the potential of this domain to facilitate SUMO binding. We first validated our MS data by immunoblotting for SEC13, SEH1L, and TAF5 and confirmed binding to SUMO2/3 WT and IKR/QFI (Fig. 7A). Then, we confirmed the direct interaction between recombinantly produced glutathione *S*-transferase (GST)–SEC13 and His^10^-SUMO2 (Fig. 7B) and GST-SEH1L and His^10^-SUMO2 using a GST–pull-down assay (Fig. 7C). This suggests that WD40 repeat domain–containing proteins might represent a class of noncovalent SUMO binders.

To decipher which part of the WD40 domains is interacting with SUMO and identify the surface on SUMO involved in this interaction, we modeled the interaction between SEC13 and SUMO2 using AlphaFold-3 (Fig. 7D and movie S1) (*32*). The predicted template modeling (pTM) score was 0.72, suggesting that the overall fold of the structure could be accurate. The relative positions of SEC13 and SUMO2 were more variable between models. Upon inspecting the individually generated models, we noted that the same specific amino acids in SEC13 contributed to the SUMO2 interaction in each of the five models, including D14-M15-H17, P59-Y79, I151, and R216-Q236. Moreover, we used WD40-repeat protein Structure Prediction database (WDSPdb) to predict potential surface hotspot residues responsible for protein-protein interactions on SEC13 and found P59, Y79, and R216 overlapping with the AlphaFold-3 models (*33*). On the basis of this, we generated a SEC13 P59A-Y79A-I151A-R216A-Q236A mutant and the following SUMO2 mutants: D25A-E48A-Q56A-R58A, N67A-E68A-T69A, D25A-E48A-Q56A-R58A-N67A-E68A-T69A, and ∆D84-G92. The C-terminal nine amino acids (84-DVFQQQTGG-92) make up the key interaction interface in three of the AlphaFold-3 models, with F86-Q87-Q88-Q89-T90 being particularly important for the interaction. We confirmed reduced binding of SUMO2 ∆D84-G92 to SEC13 WT in a GST–pull-down assay (Fig. 7E and fig. S8A). Additionally, we confirmed reduced binding of SEC13 P59A-Y79A-I151A-R216A-Q236A to SUMO2 WT (Fig. 7F and fig. S8B). This suggests that these regions are involved in the interaction between SUMO2/3 and WD40 repeat domains.

### SUMOylation of many proteins depends on noncovalent SUMO-SIM interactions

**Fig. 8.**
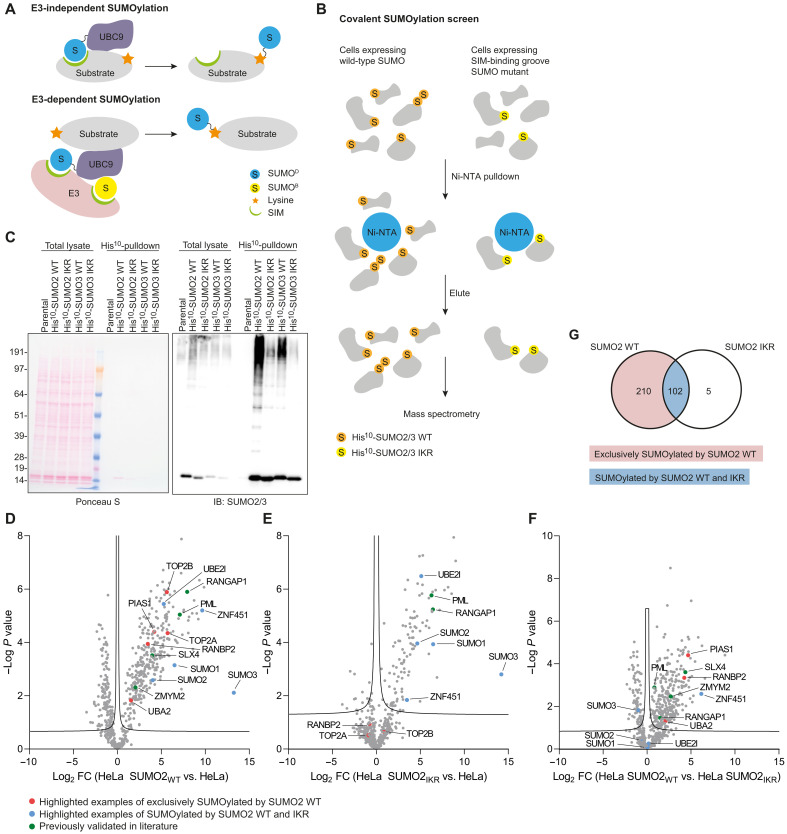
A covalent SUMOylation screen to explore SIM-dependent SUMOylation. (**A**) Cartoon illustrating a proposed model for the SIM dependency of covalent SUMOylation through the SIM-mediated recruitment of SUMO thioester-charged UBC9 to substrates, thereby facilitating lysine modification (top). Cartoon illustrating a proposed model for the SIM dependency of covalent SUMOylation through SUMO E3-dependent SUMOylation (bottom). RANBP2 contains a SIM that interacts with the donor SUMO (SUMO^D^) and anchors it in the correct orientation for discharge. ZNF451 contains two SIMs in tandem, one which anchors the donor SUMO and another which binds the backside SUMO (SUMO^B^) of UBC9. Members of the PIAS family also contain at least one SIM-like motif that positions the donor SUMO in the correct conformation. (**B**) Cartoon illustrating the experimental setup of the screen. HeLa cells expressing His^10^-SUMO2 WT or His^10^-SUMO2 IKR were lysed, and SUMOylated proteins were enriched by means of Ni-NTA pulldown, eluted, and analyzed by MS. (**C**) SUMO2/3 immunoblotting of a representative replicate of the screen, *n* = 4 independent experiments. Total lysates and His^10^–pull-down elutions were analyzed. Equal loading of total lysates was verified by Ponceau S staining. (C) Duplicated in the replicate 1 panel in fig. S9B. Volcano plots depicting the enriched proteins for HeLa His^10^-SUMO2 WT (**D**) and HeLa His^10^-SUMO2 IKR (**E**) compared to parental HeLa and for HeLa His10-SUMO2 WT compared to HeLa His^10^-SUMO2 IKR (**F**). Red dots represent highlighted examples of proteins exclusively SUMOylated in HeLa His^10^-SUMO2 WT; green dots represent proteins previously validated in literature; blue dots represent highlighted examples of proteins SUMOylated in both HeLa His^10^-SUMO2 WT and IKR. (**G**) Venn diagram for the identified SUMOylated proteins. Proteins exclusively SUMOylated by His^10^-SUMO2 WT are highlighted in red, and proteins SUMOylated by both His^10^-SUMO2 WT and IKR are highlighted in blue.

In a previous noncovalent SUMO interactor screen, close to 90% of the identified SUMO1, SUMO2, or SUMO2 trimer–binding proteins were also identified as covalent SUMO substrates (*25*). We have previously demonstrated that mutating the functional SIM in SLX4 abrogates its covalent SUMOylation (*29*). Similarly, others have shown that SUMOylation of USP25, BLM, HIPK2, and DAXX is dependent on SIMs and binding to SUMO2/3 (*28*, *34*, *35*). A proposed model for this SIM dependency is through the recruitment of SUMO thioester-charged UBC9 to these substrates, thereby facilitating lysine modification (Fig. 8A). Another possibility is through the requirement of SUMO E3 ligases to noncovalently interact with the donor SUMO through a SIM, and/or in the case of some E3 ligases also a backside binding SUMO, for efficient SUMO discharge from UBC9 followed by substrate modification (Fig. 8A). However, it is still unclear to what extent covalent SUMOylation of proteins is dependent on noncovalent SUMO interaction. To screen for this in an unbiased manner, we generated HeLa cells expressing His^10^-SUMO2/3 WT (His^10^-SUMO2/3 WT) or a SIM-binding groove SUMO2/3 mutant (His^10^-SUMO2/3 IKR). We observed a notable decrease in His^10^-SUMO2/3–conjugated proteins in cells expressing His^10^-SUMO2/3 IKR compared to His^10^-SUMO2 WT as determined by immunoblotting (fig. S9A), suggesting that mutating the SIM-binding groove of SUMO2/3 impairs its conjugation to substrates. The SIM-binding groove SUMO mutants SUMO2 QFI and SUMO3 I32A/K33A were previously shown to efficiently form thiol ester intermediates with UBC9 and SUMOylate RANGAP1 and RepoMan (*28*, *34*, *36*).

**Fig. 9.**
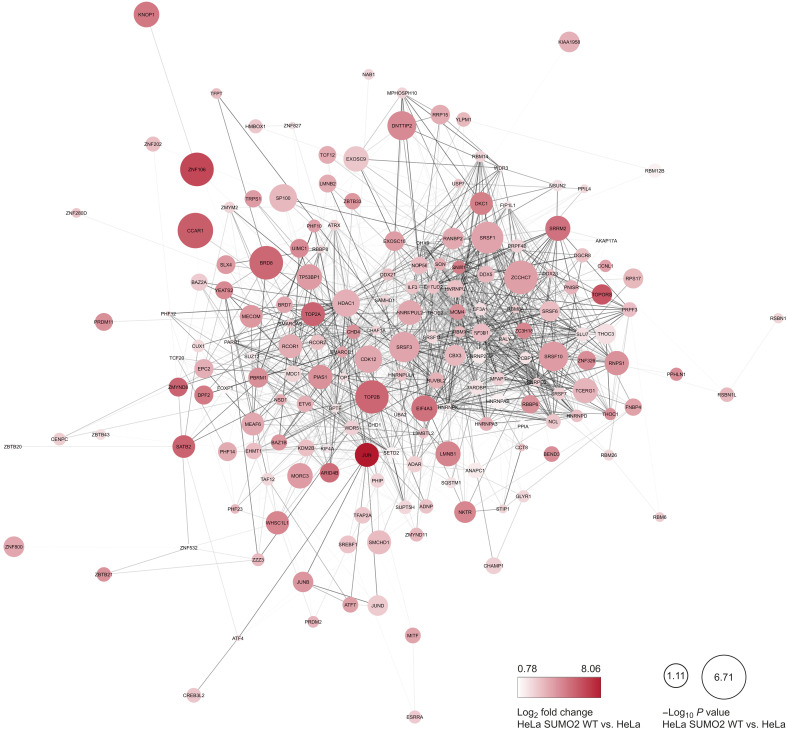
Proteins exclusively SUMOylated by SUMO2/3 WT. STRING network of proteins exclusively SUMOylated in HeLa His^10^-SUMO2 WT. Node color and size indicate the log_2_ fold change and −log *P* value, respectively, of the enriched proteins compared to parental HeLa.

**Fig. 10.**
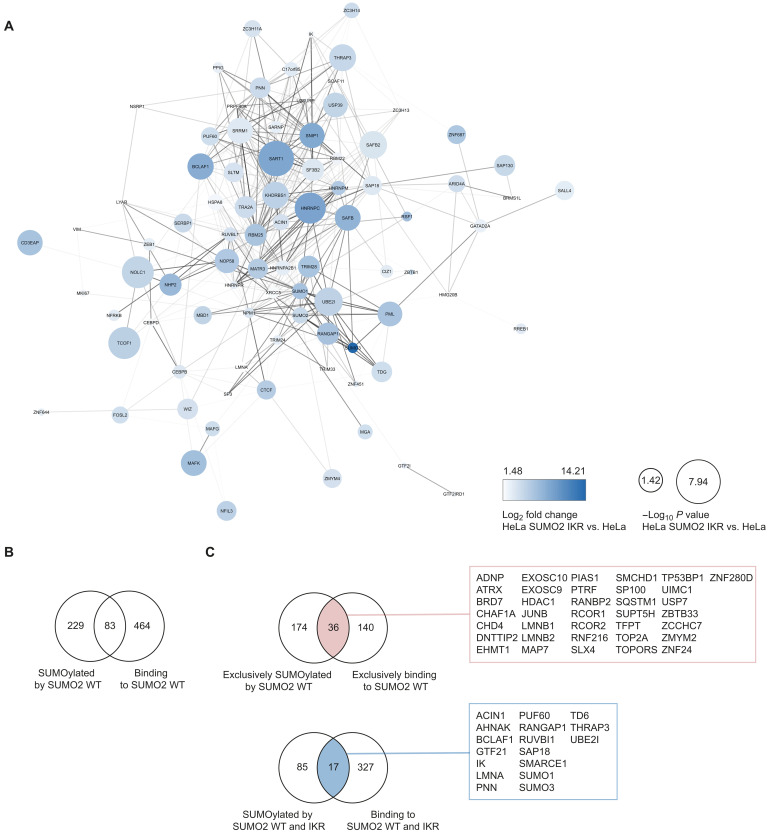
Overlap between covalent SUMOylation and noncovalent SUMO binding. (**A**) STRING network of proteins SUMOylated in both HeLa His^10^-SUMO2 WT and HeLa His^10^-SUMO2 IKR. Node color and size indicate the log_2_ fold change and −log *P* value, respectively, of the enriched proteins compared to parental HeLa. (**B**) Venn diagram of the SUMO2 binders identified in the noncovalent screen and the proteins SUMOylated by SUMO2 as identified in the covalent screen. (**C**) Venn diagram of the proteins exclusively SUMOylated by His^10^-SUMO2 WT and proteins exclusively binding to His^10^-SUMO2 WT (top). Overlapping proteins are highlighted in red and listed in the adjacent box. Venn diagram of the proteins SUMOylated by both His^10^-SUMO2 WT and IKR, and proteins binding to both His^10^-SUMO2 WT and IKR (bottom). Overlapping proteins are highlighted in blue and listed in the adjacent box.

SUMOylated proteins in HeLa His^10^-SUMO2/3 WT and His^10^-SUMO2/3 IKR were enriched by means of Ni-NTA pull-down and analyzed by MS (Fig. 8B). Four biological replicates were performed. Immunoblotting for SUMO2/3 confirmed efficient enrichment of His^10^-SUMOylated proteins (Fig. 8C and fig. S9B). After removing common contaminants and non-consistently identified proteins, further analysis was performed for 636 and 583 protein groups for SUMO2 (table S3) and SUMO3 (table S4), respectively. Heatmap analysis (fig. S10A) and PCA clustering (fig. S10B) of the identified proteins showed clustering of biological replicates by experimental condition, indicating high reproducibility. We identified 312 SUMOylated proteins in HeLa cells expressing His^10^-SUMO2 WT and 107 SUMOylated proteins in cells expressing His^10^-SUMO2 IKR (FDR < 0.05, *s0* = 0.1) (Fig. 8, D and E). This difference is in line with the observed reduction in His^10^-SUMO2 conjugates described above. Because of the large overlap in identified proteins for HeLa SUMO2 and SUMO3 (only nine unique proteins identified in HeLa SUMO3 WT and one unique protein in HeLa SUMO3 IKR), we focused the analysis on HeLa SUMO2. Of the 312 SUMOylated proteins in HeLa cells expressing His^10^-SUMO2 WT, 102 proteins were also identified as SUMOylated proteins in HeLa cells expressing His^10^-SUMO2 IKR. Comparing His^10^-SUMO2 WT with His^10^-SUMO2 IKR, 210 proteins were exclusively identified in HeLa His^10^-SUMO2 WT cells and 256 proteins showed preferential enrichment in His^10^-SUMO2 WT cells (FDR < 0.05, *s0* = 0.1) (Fig. 8, F and G), representing proteins whose SUMOylation is dependent or is at least promoted by noncovalent interaction with SUMO. A smaller selection of proteins showed equal enrichment for His^10^-SUMO2 WT and His^10^-SUMO2 IKR, suggesting that the reduction in SUMO-conjugated proteins for His^10^-SUMO2 IKR is not merely a result of a global reduction in the SUMOylation levels of all SUMOylated proteins but is rather a consequence of substrate-specific reductions in SUMOylation. A total of 210 proteins were exclusively SUMOylated by His^10^-SUMO2 WT, while 102 proteins were SUMOylated by both His^10^-SUMO2 WT and IKR (Fig. 8G). We performed STRING analysis on the proteins exclusively SUMOylated by His^10^-SUMO2 WT (Fig. 9) and the proteins SUMOylated by both His^10^-SUMO2 WT and IKR (Fig. 10A).

Supporting the validity of our screen, we could confirm the previously described SIM dependency of SLX4 SUMOylation, with SLX4 being significantly enriched in cells expressing His^10^-SUMO2 WT and not in cells expressing His^10^-SUMO2 IKR (Fig. 8, D to F). Moreover, noncovalent binding of ZMYM2 to SUMO was previously shown to require the SIM-binding groove of SUMO (*24*, *27*), and we show that this is also required for its covalent SUMOylation (Fig. 8, D to F). In contrast, promyelocytic leukemia protein (PML) is still SUMOylated when its SIM domain is mutated (*37*), which is in line with our observation that SUMOylated PML was also significantly enriched in cells expressing His^10^-SUMO2 IKR (Fig. 8, D to F). In vitro SUMOylation of RANGAP1 does not involve noncovalent interaction with SUMO and, accordingly, was significantly enriched in cells expressing His^10^-SUMO2 IKR (Fig. 8, D to F). Together, these data suggest that many proteins require noncovalent binding to the SIM-binding groove of SUMO for their covalent SUMOylation, while proteins also exist not showing this dependency. This suggests that the covalent SUMOylation of these proteins is regulated by distinct mechanisms.

### SUMOylation of the Ub/Ubl machinery in context of noncovalent SUMO interactions

Focusing on proteins from the Ub/Ubl machinery identified in our dataset, we observed differential enrichment levels for the SUMO E3 ligases PIAS1, RANBP2, and ZNF451 and SUMO-activating enzyme subunit UBA2 between His^10^-SUMO2 WT and IKR, although ZNF451 was also SUMOylated by His^10^-SUMO2 IKR (Fig. 8, D to F). In contrast, the SUMO paralogs SUMO1, SUMO2, and SUMO3 and the SUMO-conjugating enzyme UBC9 were enriched to similar levels, suggesting that covalent SUMOylation of these proteins is independent of noncovalent interactions with SUMO.

### Overlap between covalent SUMOylation and noncovalent SUMO binding

Of the 312 SUMOylated proteins in His^10^-SUMO2 WT cell lines, only 83 proteins were identified as noncovalent SUMO2 WT interactors (Fig. 10B). In other words, most of proteins SUMOylated by SUMO2 WT did not bind to SUMO2 WT (73.4%), suggesting that the covalent SUMOylation of many proteins does not require direct noncovalent interaction of these proteins but perhaps depends on other proteins interacting with the SIM-binding groove of SUMO, such as SUMO E3 ligases. Of the 210 proteins that were exclusively SUMOylated by His^10^-SUMO2 WT and not IKR, 36 proteins also exclusively bound to SUMO2 WT. These could be strong candidates for direct SUMO-SIM interaction–dependent SUMOylation of the protein (Fig. 10C). Of the 102 proteins SUMOylated by both His^10^-SUMO2 and His^10^-SUMO2 IKR, 17 proteins also bound to both SUMOs (Fig. 10C).

## DISCUSSION

We are limited in our knowledge on noncovalent SUMO interactions beyond the typical SUMO-SIM interaction. Our screen allows for both the selection of proteins that bind through class I SUMO-SIM interactions with higher confidence (the “exclusive” binders) and provides a resource of proteins binding to SUMO through other modes of interaction (the “common” binders). We found that many proteins were able to bind to both SUMO2/3 WT and the SIM-binding groove mutant SUMOs. Of these, only a small number bound preferentially to SUMO WT, suggesting that the binding has a binary nature. The common binders represent proteins that do not bind to the SIM-binding groove of SUMO but rather to another region and not through a conventional SIM. Thus, the prevalence and relevance of SUMO-SIM–independent modes of SUMO interaction is now underestimated. We also identified proteins binding exclusively to the SIM-binding groove mutants and not to SUMO WT. These were, therefore, excluded from downstream analyses. It is unclear whether mutating the SIM-binding groove creates a different interaction surface or whether this is a consequence of experimental design; one possible explanation could be that the recombinant SUMO was limiting on the beads, causing SIM-binding proteins in the lysate binding to SUMO WT to saturate the beads, thereby limiting the binding of proteins interacting with other SUMO surfaces.

So far, only one other SUMO interactomics study was designed to specifically capture proteins that use alternative modes of SUMO interaction, rather than capturing all SUMO interactors irrespective of the mode of interaction. In this screen, a photocrosslinking approach was used to enrich for class II SUMO1–interacting proteins (*19*). In our previous SUMO interactome study, we show that most of SUMO2 binders are unique SUMO2 binders that do not bind to SUMO1, while only a handful of SUMO1 binders bind exclusively to SUMO1 and not to SUMO2 (*25*). Besides UBC9, we find 22 proteins overlapping with the identified class II SUMO1 interactors. These could be proteins that interact with SUMO via class II binding, in a paralog-independent manner. One of the identified proteins is valosin-containing protein (VCP), an AAA^+^ ATPase described to regulate the extraction of SUMO-and ubiquitin-modified proteins from protein complexes and other cellular structures. We have previously identified VCP as a SUMO1, SUMO2, and tri-SUMO2 binder in a similar screening approach (*25*). Recently, VCP was also shown to bind to polySUMO2 chains in a proteome microarray screening (*22*).

Using domain enrichment analysis, we identified several domains enriched among the proteins interacting exclusively with SUMO WT and both SUMO WT and the SIM-binding groove mutants. Zinc finger proteins exclusively bound to SUMO WT and not to the SIM-binding groove mutants, which is in agreement with previous studies describing interaction of zinc fingers with SUMO through the SIM-binding groove (*24*, *27*). Among the domains enriched among the proteins capable of binding to the SIM-binding groove mutants, a group of WD40 repeat domain–containing proteins was of specific interest. WD40 repeat domains are ß-propellor domains and proteins typically consist of seven ß-propellor repeats. WD40 domains are the most abundant protein interaction domain in the human proteome and function as interaction scaffolds in protein complexes in a variety of cellular processes. WD40 repeat domain proteins were described to bind ubiquitin in yeast and bacteria (*31*). Here, we show that a selection of WD40 repeat domain proteins also bind to SUMO. We confirmed direct interaction in vitro between SEC13 and SUMO2 and between SEH1L and SUMO2. Because some of these proteins consist almost entirely of the WD40 repeat domain, we expect this domain to participate in the interaction with SUMO.

Using structural modeling of the SEC13 interaction with SUMO using AlphaFold-3 followed by experimental validation of SEC13 and SUMO2 mutants, we identified residues in the WD40 domain of SEC13 and SUMO2 C terminus involved in the interaction. SEC13 consists of six ß-propellor repeats. Each ß-propellor repeat in WD40 domains folds into a four-strand anti-paralleled β sheet—strands d, a, b, and c—connected by loops. Most of the known WD40 interactions are focused on the top face of the WD40 ß-propellor, formed by the loops connecting d-a and b-c strands. The P59, Y79, I151, and Q236 residues that we identified in SEC13 locate to the top face of the ß-propellor in loops connecting d-a and b-c strands in repeat 2, 4, and 5, while R216 locates to strand a in repeat 5. Y79, R216, and Q236 were also predicted as mutational hotspots for protein-protein interactions in WDSPdb (*33*). Collectively, this supports that these regions are involved in the interaction between WD40 repeat domains and SUMO2/3, although individual residues might differ between WD40 repeat domain proteins. The WD40 repeat domain proteins comprise around 8% of the proteins binding to the SIM-binding groove mutants, suggesting that the other proteins that were identified might interact through yet uncharacterized binding domains or sequence motifs. Approaches such as nuclear magnetic resonance spectroscopy could further aid in the identification of residues involved in the interaction in both the interaction partner and SUMO.

One of the protein clusters enriched in both the exclusive SUMO2 binders as well as the common SUMO2 binders was protein SUMOylation. We confirmed UBC9 as one of the common interactors and also identified the SUMO E1 enzyme SAE1/UBA2 and the SUMO proteases SENP1-2 and SENP5. Other SUMO machinery components do seem to require the SIM-binding groove for binding. As expected, the SUMO-targeted ubiquitin ligases RNF4 and RNF111, which are known to consist of multiple SIMs, exclusively bind to the SIM-binding groove of SUMO (*38*, *39*). Recently, a multi-SIM domain in the N-terminal region of SENP6 was shown to be required for SUMO binding, specifically polymeric SUMO chains (*40*). We confirm that SENP6 requires the SIM-binding groove of SUMO for noncovalent interaction. We also identified the SUMO E3 ligases PIAS1-4, ZNF451, TOPORS, and RANBP2 as exclusive SUMO2 WT interactors. These ligases contain at least one functional SIM, and this is pivotal for their catalytic activity by participating in the interaction with SUMO-charged UBC9 [(*41*–*43*), reviewed in (*6*)]. This could explain the lower levels of covalent SUMO modification in cells expressing SUMO IKR, rendering the SUMO E3 ligases less effective in stimulating the discharge of SUMO from the SUMO-UBC9 thioester. This contrasts with in vitro experiments, where SUMO E3 ligases are not required for SUMO conjugation. Another factor contributing to the lower levels of SUMO modification in cells expressing SUMO IKR could be impaired recruitment of SUMO thioester-charged UBC9 to substrates (*5*). Proteins that are still SUMOylated in cells expressing SUMO2 IKR perhaps do not require the aid of a SUMO E3 ligase or noncovalent interaction with the SUMO moiety of SUMO thioester-charged UBC9 for their covalent SUMOylation. By performing an unbiased screen with a SIM-binding groove SUMO mutant, we can assess on a larger scale which SUMO targets are potentially affected through these established mechanisms. Together, our dataset is a unique resource and offers valuable insights on the intricacies of the SUMO interactome. Additionally, we extended our understanding of the SUMO interactome to also include covalent SUMO modification in the context of the SIM-binding groove.

## MATERIALS AND METHODS

All antibodies, oligonucleotides, and recombinant DNA used are listed in table S5.

### Cell culture

HeLa and human embryonic kidney (HEK) 293 T cells were cultured in Dulbecco’s modified Eagle’s medium (DMEM) (high glucose, pyruvate, Gibco) supplemented with 10% fetal calf serum and penicillin (100 U/ml) and streptomycin (100 μg/ml; Gibco). Cells were checked routinely for mycoplasma and confirmed to be negative.

### Generating SIM-binding groove SUMO mutants

Mature SUMO2 and SUMO3 were annotated according to the original literature and not the databases. SUMO2 (UniProt, P55854): 1-MSEEKPKEGVKTENDHINLKVAGQDGSVVQFKIKRHTPLSKLM-KAYCERQGLSMRQIRFRFDGQPINETDTPAQLEMEDEDTIDVF-QQQTGG-92. SUMO3 (UniProt, P61956): 1-MADEKPKEGVKTE-NNDHINLKVAGQDGSVVQFKIKRHTPLSKLMKAYCERQGLSMRQIRFRFDGQPINETDTPAQLEMEDEDTIDVFQQQTGG-93. pUC57-His^10^-SUMO2 WT and pUC57-His^10^-SUMO3 WT were synthesized and obtained from GenScript. The SIM-binding groove on SUMO2 and SUMO3 was abrogated by mutating I33-K34-R35 to alanine residues (SUMO2/3 IKR) or Q30-F31-I33 to alanine residues (SUMO2/3 QFI) using two-step polymerase chain reaction–mediated mutagenesis. The following primers were used. SUMO2 IKR: 5′-GCTCCGTGGTGCAGTTCAAGgcCgc-GgcGCACACGCCGCTGAGCAAGCT-3′ (forward) and 5′-AGCT-TGCTCAGCGGCGTGTGCgcCgcGgcCTTGAACTGCACCACGGAGC-3′ (reverse); SUMO3 IKR: 5′-GTTCTGTGGTGCAGTTTA-AGgcTgcGgcGCATACACCACTTAGTAAACT-3′ (forward) and 5′-AGTTTACTAAGTGGTGTATGCgcCgcAgcCTTAAACTGCACC-ACAGAAC-3′ (reverse); SUMO2 QFI: 5′-GGCAGGACGGCTCCGTGGTGGCGGCCAAGGCCAAGAGGCACACGCCGCGAG-3′ (forward) and 5′-CTCAGCGGCGTGTGCCTCTTGGCCTTGGCCGCCACCACGGAGCCGTCCTGCC-3′ (reverse); and SUMO3 QFI: 5′-GGCAGGATGGTTCTGTGGTGGCGGCTAAGGCTAAGAGGCATACACCACTTAG-3′ (forward) and 5’-CTAAGTGGTGTAT-GCCTCTTAGCCTTAGCCGCCACCACAGAACCATCCTGCC-3′ (reverse). Mutants were verified by sequencing the entire insert.

### Generating SUMO-SEC13 interaction mutants

pUC57 encoding His^10^-SUMO2 D25A-E48A-Q56A-R58A, N67A-E68A-T69A, D25A-E48A-Q56A-R58A-N67A-E68A-T69A, and ∆D84-G92 sites was synthesized and obtained from GenScript. pDONR207 encoding SEC13 P59A-Y79A-I151A-R216A-Q236A was synthesized and obtained from GenScript.

### Recombinant protein production and purification

His^10^-SUMO2 and SUMO3 WT/QFI/IKR/D25A-E48A-Q56A-R58A/N67A-E68A-T69A/D25A-E48A-Q56A-R58A-N67A-E68A-T69A/∆D84-G92 were cloned into pET-11a-UBC9-His^10^ (GenScript) using restriction enzyme cloning. pUC57 encoding the His^10^-SUMO2/3 proteins was digested with Bam HI and Nde I and ligated into pET-11a-UBC9-His^10^ linearized with Eco RI and Not I, thereby replacing UBC9-His^10^ with the His^10^-SUMO2/3 proteins. The His^10^-SUMO2/3 proteins were recombinantly expressed in Rosetta *E. coli* grown to an optical density (OD) at 600 nm of ~0.6 to 0.8 at 37°C. Protein expression was induced with 0.5 mM isopropyl-β-d-thiogalactopyranoside (IPTG) for 6 hours at 30°C. Bacteria were lysed in 50 mM Hepes (pH 7.6), 0.5 M NaCl, 50 mM imidazole (pH 7.0), and 200 mM phenylmethanesulfonylfluoride (PMSF) by sonication. The His^10^-SUMO proteins were purified from the lysates by gravity-flow Ni-NTA bead purification. Beads were washed twice with 50 mM Hepes (pH 7.6), 0.5 M NaCl, and 50 mM imidazole (pH 7.0). Proteins were eluted from the beads with 50 mM Hepes (pH 7.6), 0.5 M NaCl, and 500 mM imidazole (pH 7.0). Purification was verified by gel electrophoresis and colloidal blue staining. SEC13 was cloned from pDONR223 (Sigma-Aldrich, MISSION TRC3 Human ORF Collection) into pDEST15 using gateway cloning. A stop codon was first introduced in pDONR223-SEC13 using site-directed mutagenesis. SEC13 P59A-Y79A-I151A-R216A-Q236A mutant was also cloned from pDONR207 into pDEST15 using gateway cloning. SEH1L was cloned from pRK5 (Addgene, no. 154216) into pDONR207 and then into pDEST15 using gateway cloning. GST-SEC13 and GST-SEH1L were recombinantly expressed in Rosetta grown to an OD at 600 nm of ~0.6 to 0.8 at 37°C. Protein expression was induced with 1 mM IPTG for 3 to 4 hours at 37°C. Bacteria were lysed in ice-cold phosphate-buffered saline (PBS), 0.5 M NaCl, 1 mM PMSF, and one tablet of cOmplete Protease Inhibitor Cocktail (Sigma-Aldrich) per 10 ml of buffer by sonication. Purification was verified by gel electrophoresis and colloidal blue staining. Lysates were stored at −20°C until further processing for in vitro SUMO binding.

### Generating His^10^-SUMO–expressing cell lines

His^10^-SUMO2 and SUMO3 WT/IKR were cloned into pLV-IRES-puro using restriction enzyme cloning. pUC57 encoding the His^10^-SUMO2/3 proteins was digested with Xho I and Pst I and ligated into pLV-IRES-puro linearized with Xho I and Pst I. Lentivirus was produced in HEK 293 T cells transfected with third-generation lentiviral packaging plasmids (pCMV-VSVG, pMDLg-RRE, and pRSV-REV) and the pLV plasmids encoding His^10^-SUMO2 and His^10^-SUMO3 WT/IKR. HeLa were transduced with the harvested lentivirus in DMEM containing polybrene (8 μg/ml). Transduced cells were selected in medium containing puromycin (1 μg/ml). Expression of His^10^-SUMO2 and SUMO3 WT/IKR was confirmed by His and SUMO immunoblotting.

### In vitro SUMO binding screen

Recombinant His^10^-tagged SUMO2 and SUMO3 proteins were produced as described above. Ni-NTA bead slurry (700 μl) was washed three times with 50 mM tris (pH 7.5), 150 mM NaCl, 0.5% NP-40, and 50 mM imidazole (pH 7.0) and split over seven 100-μl aliquots. To six aliquots, 100 μg of recombinant His^10^-SUMO2 WT, His^10^-SUMO2 IKR, His^10^-SUMO2 QFI, His^10^-SUMO3 WT, His^10^-SUMO3 IKR, or His^10^-SUMO3 QFI was added; the remaining aliquot of beads was used as negative control. Subsequently, beads were incubated for 2 hours at 4°C while rotating and then washed again three times with 50 mM tris (pH 7.5), 150 mM NaCl, 0.5% NP-40, and 50 mM imidazole (pH 7.0).

Thirty-five confluent 15-cm dishes of HeLa cells were lysed in 7 ml of lysis buffer [50 mM tris (pH 7.5), 150 mM NaCl, 0.5% NP-40, and 50 mM imidazole (pH 7.0)]. Lysates were sonicated two times for 10 s, split over 1.5-ml microcentrifuge tubes, and centrifuged for 1 hour at 4°C at 16,000*g*. Input samples were taken from the supernatant, and the remaining supernatant was divided over the Ni-NTA beads with the His^10^-SUMO2/3 proteins and beads only and incubated for 2 hours at 4°C while rotating. Bicinchoninic acid (BCA) Protein Assay Reagent (Thermo Fisher Scientific) was used to determine protein concentration. After incubation, an unbound control sample was taken, and beads were washed three times for 10 min with lysis buffer [50 mM tris (pH 7.5), 150 mM NaCl, 0.5% NP-40, and 50 mM imidazole (pH 7.0)], followed by three times for 10 min with wash buffer [50 mM tris (pH 7.5) and 150 mM NaCl], including a tube change after each second wash. Proteins were eluted from the beads with 100 μl of elution buffer 1: 8 M urea and 50 mM tris (pH 7.5) for 30 min at 4°C at 1200 rpm. The second elution was performed with 100 μl of elution buffer 2: 8 M urea, 50 mM tris (pH 7.5), and 500 mM imidazole (pH 7.0) for 30 min at 4°C at 1200 rpm. Elutions were separated from any remaining beads using a prewashed 0.45-μm filter column (Millipore) and centrifugation at 10,000*g*. Small aliquots of each elution were kept for immunoblotting.

Samples from the first elution were concentrated using a 50-kDa cutoff filter prewashed with elution buffer 1. Concentration was performed by centrifugation at 8000*g* at room temperature. Next, the proteins were washed once with 250 μl of elution buffer and eluted from the filter. Samples were equalized using elution buffer 1. Ammonium bicarbonate (ABC) was added to a concentration of 50 mM. Dithiothreitol (DTT) was added to a concentration of 1 mM and incubated for 30 min at room temperature. Then, chloroacetamide (CAA) was added to a concentration of 5 mM and incubated for 30 min at room temperature, followed by adding DTT to 5 mM and incubating for 30 min at room temperature. Samples were diluted fourfold by adding three volumes of 50 mM ABC. Trypsinization was performed by adding 250 ng of trypsin and digesting overnight, still and in the dark.

### Purification of His^10^-SUMOylated proteins from cells

His^10^-SUMO2– and His^10^-SUMO3–conjugated proteins were purified as described previously (*44*). In brief, HeLa cells stably expressing His^10^-SUMO2 proteins or His^10^-SUMO3 proteins were harvested in ice-cold PBS. For total protein lysates, a small fraction of cells was lysed in 2% SDS, 1% NP-40, 50 mM tris (pH 7.5), and 150 mM NaCl. The remainder was lysed in 25 pellet volumes of 6 M guanidine-HCl, 100 mM Na_2_HPO_4_/NaH_2_PO_4_, and 10 mM tris, buffered at pH 8.0. Lysates were snap-frozen in liquid nitrogen and stored at −80°C or directly processed. Cells were thawed at room temperature and sonicated twice for 10 s at 30 W. Protein concentration was determined by BCA, and lysates were equalized. Subsequently, lysates were supplemented with 5 mM β-mercaptoethanol and 50 mM imidazole (pH 8.0). Prewashed Ni-NTA beads (QIAGEN, 30210) were added to the lysates and incubated overnight at 4°C. Ni-NTA beads were washed with wash buffer 1 to buffer 4, respectively; Wash buffer 1: 6 M guanidine-HCl, 100 mM sodium phosphate, 10 mM tris, 10 mM imidazole, 5 mM β-mercaptoethanol, and 0.2% Triton X-100. Wash buffer 2: 8 M urea, 100 mM sodium phosphate, 10 mM tris, 10 mM imidazole, 5 mM β-mercaptoethanol, and 0.2% Triton X-100. Wash buffer 3: 8 M urea, 100 mM sodium phosphate, 10 mM tris, 10 mM imidazole, 5 mM β-mercaptoethanol, and 0.2% Triton X-100. Wash buffer 4: 8 M urea, 100 mM sodium phosphate, 10 mM tris, 5 mM β-mercaptoethanol, and 0.1% Triton X-100. Purified proteins were eluted thrice in one bead volume of 7 M urea, 100 mM sodium phosphate, 10 mM tris, and 500 mM imidazole (pH 7.0).

Elutions were separated from any remaining beads using a prewashed 0.45-μm filter column (Millipore) and centrifugation at 10,000*g*. Elutions were concentrated using a 100-kDa cutoff filter prewashed with elution buffer. Concentration was performed by centrifugation at 8000*g* at room temperature. Next, the proteins were washed once with 250 μl of elution buffer and eluted from the filter. Samples were equalized using elution buffer. ABC was added to a concentration of 50 mM. DTT was added to a concentration of 1 mM and incubated for 30 min at room temperature. Then, CAA was added to a concentration of 5 mM and incubated for 30 min at room temperature, followed by adding DTT to 5 mM and incubating for 30 min at room temperature. Samples were diluted fourfold by adding three volumes of 50 mM ABC. Trypsinization was performed by adding 250 ng of trypsin and digesting overnight, still and in the dark.

### Stage tipping

Trypsin-digested peptides were desalted and concentrated on stage tips as described previously and eluted with 50% acetonitrile in 0.1% formic acid (*45*). Elutions were vacuum dried in a SpeedVac and dissolved in 10 μl of 0.1% formic acid before nanoflow liquid chromatography–tandem MS (LC-MS/MS).

### LC-MS/MS analysis

Samples were analyzed by online C18 nano HPLC MS/MS with a system consisting of an UltiMate 3000 nano gradient HPLC system (Thermo Fisher Scientific, Bremen, Germany) and an Exploris480 mass spectrometer (Thermo Fisher Scientific, Bremen, Germany). Digested peptides were injected onto a cartridge precolumn (300 μm by 5 mm, C18 PepMap, 5 um) in 100% solvent A (0.1% Milli-Q water), with a flow of 250 nl/min for 3 min (Thermo Fisher Scientific, Bremen, Germany) and eluted via a homemade analytical nano HPLC column (30 cm by 75 μm; Reprosil-Pur C18-AQ 1.9 μm, 120 A (Dr. Maisch, Ammerbuch, Germany).

For the noncovalent SUMO interaction screen, the chromatography gradient length was 120 min from 2 to 36% solvent B, followed by a 1 min increase to 80% solvent B, another 5 min of 80% solvent B, and back to 2% solvent B for chromatography column reconditioning. The mass spectrometer was operated in positive polarity data–dependent MS/MS mode with a cycle time between master scans of 3 s. Full-scan MS spectra were obtained with a resolution of 120,000, a normalized automatic gain control (AGC) target set to standard, and a scan range of 300 to 1500 mass/charge ratio (*m*/*z*). Precursors were fragmented by higher-collisional dissociation (HCD) with a normalized collision energy of 30%. MS/MS spectra were recorded with a resolution of 30,000 and a normalized AGC target value of 100%. Precursor ions selected for MS/MS analysis were subsequently dynamically excluded from MS/MS analysis for 45 s and only precursors with a charge state of two to six triggered MS/MS events.

For the covalent SUMOylation screen, the chromatography gradient length was 60 min from 2 to 40% solvent B, followed by a 5 min increase to 95% solvent B, another 5 min of 95% solvent B, and back to 2% solvent B for chromatography column reconditioning. The mass spectrometer was operated in positive polarity data–dependent MS/MS mode with a cycle time between master scans of 3 s. Full-scan MS spectra were obtained with a resolution of 60,000, a normalized AGC target of 300%, and a scan range of 350 to 1600 *m*/*z*. Precursors were fragmented by HCD with a normalized collision energy of 28%. MS/MS spectra were recorded with a resolution of 30,000 and a normalized AGC target value of 75%. Precursor ions selected for MS/MS analysis were subsequently dynamically excluded from MS/MS analysis for 30 s and only precursors with a charge state of two to six triggered MS/MS events.

### MaxQuant data analysis

Raw data were analyzed using MaxQuant (version 2.0.1.0 for the covalent SUMOylation screen and version 2.1.3.0 for the noncovalent SUMO-binding screen). We performed the search against an in silico digested UniProt human reference proteome (9 June 2022 for the covalent SUMOylation screen; 27 July 2022 for the noncovalent SUMO-binding screen). Database searches were performed according to standard settings with the following modifications. Digestion with trypsin/P was used, allowing two missed cleavages. Label-free quantification (LFQ) was enabled, with fast LFQ. Oxidation (M) and acetyl (protein N-term) were allowed as variable modifications and carbamidomethyl (C) as a fixed modification with a maximum number of 5. Match between runs was performed with 0.7-min match time window and 20-min alignment time window, and match unidentified features was performed. All peptides were used for protein quantification. All tables were written.

### Perseus data analysis

Output from MaxQuant analysis was analyzed using Perseus (version 1.6.15.0). LFQ intensity values were log_2_ transformed, and potential contaminants and proteins identified only by site or reverse peptide were removed. Replicates of the same experimental conditions were grouped. Data were filtered for protein groups that had at least three valid values in at least one group. Missing values were imputed using normally distributed values with a 1.8 downshift (log_2_) and a randomized 0.3 width (log_2_) based on the total data matrix. Student’s *t* tests with a permutation-based FDR = 0.05 and an *s0* = 0.1 were performed.

### STRING, GO, and GPS-SUMO analysis

STRING, MCODE clustering, and GO enrichment analysis were performed in Cytoscape 3.9.1. STRING network analysis was performed with the default cutoff of 0.4. The MCODE plugin was used for clustering analysis using default settings. GO enrichment analysis was performed for GO biological processes and GO molecular function. Redundant terms were excluded using a cutoff of 0.1. Prediction of SIMs was performed in GPS-SUMO 1.0 with the SUMO interaction threshold set to high to filter for only the highest probability SIMs.

### Electrophoresis and immunoblotting

Cells were lysed in 2% SDS, 1% NP-40, 50 mM tris (pH 7.5), and 150 mM NaCl, unless stated otherwise. Proteins were separated on 4 to 12% gradient gels (Bold, Thermo Fisher Scientific) using Mops buffer (NuPage, Thermo Fisher Scientifc) using tris-acetate buffer. Proteins were subsequently transferred onto Amersham Protran Premium 0.45 NC Nitrocellulose blotting membrane (GE Healthcare), using a submarine system. Membranes were stained with Ponceau S to visualize total protein and blocked with PBS containing 0.05% Tween 20 and 8% milk powder. Subsequently, membranes were stained with primary and secondary antibodies diluted in PBS containing 0.05% Tween 20 and 8% milk powder.

### Computational modeling of SUMO-SEC13 interaction

Human SEC13 (UniProt, P55735) was folded together with SUMO2 (using the misannotated SUMO3 from UniProt, P55854, but omitting the C-terminal VPESSLAGHSF) using AlphaFold-3 with standard settings (*32*). The pTM score was 0.72, suggesting that the overall fold of the structure could be accurate. The relative positions of SEC13 and SUMO2 were more variable between models. Upon inspecting the individual generated models using ChimeraX, we noted that the same specific amino acids in SEC13 contributed to the SUMO2 interaction in each of the five models, including D14-M15-H17, P59-Y79, I151, and R216-Q236. In SUMO2, the C-terminal nine amino acids (84-DVFQQQTGG-92) make up the key interaction interface, with F86-Q87-Q88-Q89-T90 being particularly important for the interaction.

### In vitro SUMO binding

GST-SEC13 and GST-SEH1L bacterial lysates were bound to Glutathione Sepharose beads (GE Healthcare) in PBS, 0.5 M NaCl, 1 mM PMSF, and one tablet of cOmplete Protease Inhibitor Cocktail (Sigma-Aldrich) per 10 ml of buffer. Recombinant GST was included as control. Beads were incubated for 2 hours at 4°C while rotating. Beads were washed three times with PBS and 0.5 M NaCl and once with interaction buffer [50 mM tris (pH 7.5), 150 mM NaCl, 0.5% NP40, 5 mM imidazole (pH 7.0), and bovine serum albumin (0.1 mg/ml)]. Recombinant His^10^-SUMO2 was added to the mixture and incubated for 2 hours at 4°C while rotating. Beads were washed five times with interaction buffer. Proteins bound to the beads were eluted in 2× LDS sample buffer and 1 mM DTT. Elutions were analyzed by immunoblotting.

## Supplementary Materials

### The PDF file includes:

Figs. S1 to S10 Legends for tables S1 to S4 Table S5 Legend for movie S1 References
