# Supplementary material for: Interaction networks of SIM-binding groove mutants reveal alternate modes of SUMO binding and profound impact on SUMO conjugation
Source: Sci Adv. 2025 May 14;11(20):eadp2643. doi: 10.1126/sciadv.adp2643 (PMC12077520; doi:10.1126/sciadv.adp2643)
Supplement: Supplementary file 1 — Figs. S1 to S10 Legends for tables S1 to S4 Table S5 Legend for movie S1 References [file sciadv.adp2643_sm.pdf]

Science Advances
AAAS

Supplementary Materials for

Interaction networks of SIM-binding groove mutants reveal alternate modes of SUMO binding and profound impact on SUMO conjugation

Laura A. Claessens *et al.*

Corresponding author: Alfred C. O. Vertegaal, vertegaal@lumc.nl

Sci. Adv. **11**, eadp2643 (2025)
DOI: 10.1126/sciadv.adp2643

The PDF file includes:

Figs. S1 to S10
Table S5
Legends for tables S1 to S4
Legend for movie S1
References

Other Supplementary Material for this manuscript includes the following:

Tables S1 to S4
Movie S1

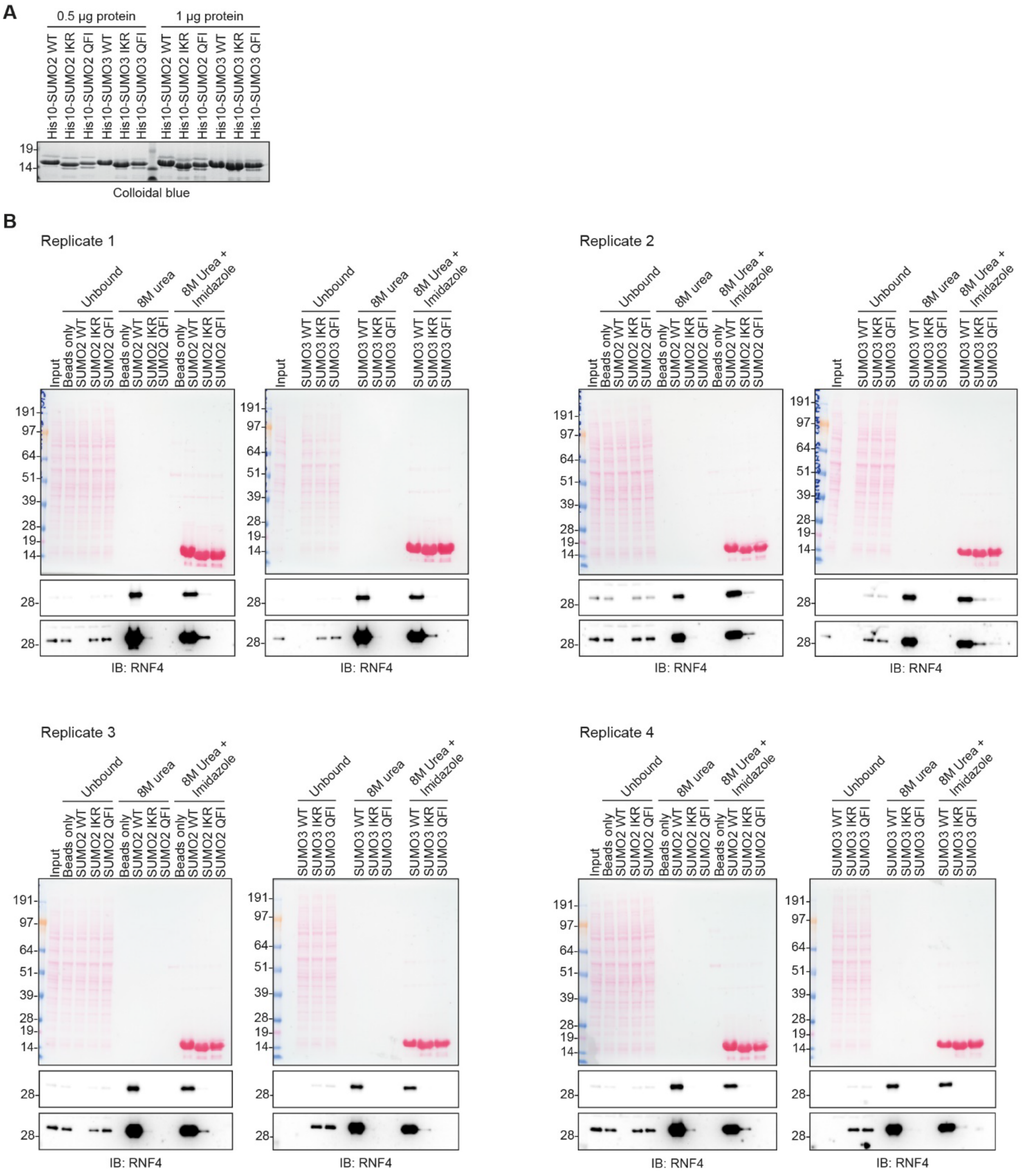

Supplementary Figure 1. Immunoblotting corresponding to Figure 1. (A) Colloidal blue staining of the recombinant proteins used in the noncovalent interaction screen, as described in Figure 1A-B. Two amounts were loaded to visualize the proteins. **(B)** Immunoblotting of all replicates of the noncovalent interaction screen, n=4 independent experiments (top: short exposure; bottom: long exposure). Anti-RNF4 was used as a control for binding to SUMO2/3 wildtype, but not the SIM-binding groove mutants. Replicate 1 in Panel B is duplicated from Main Figure 1 Panel C.

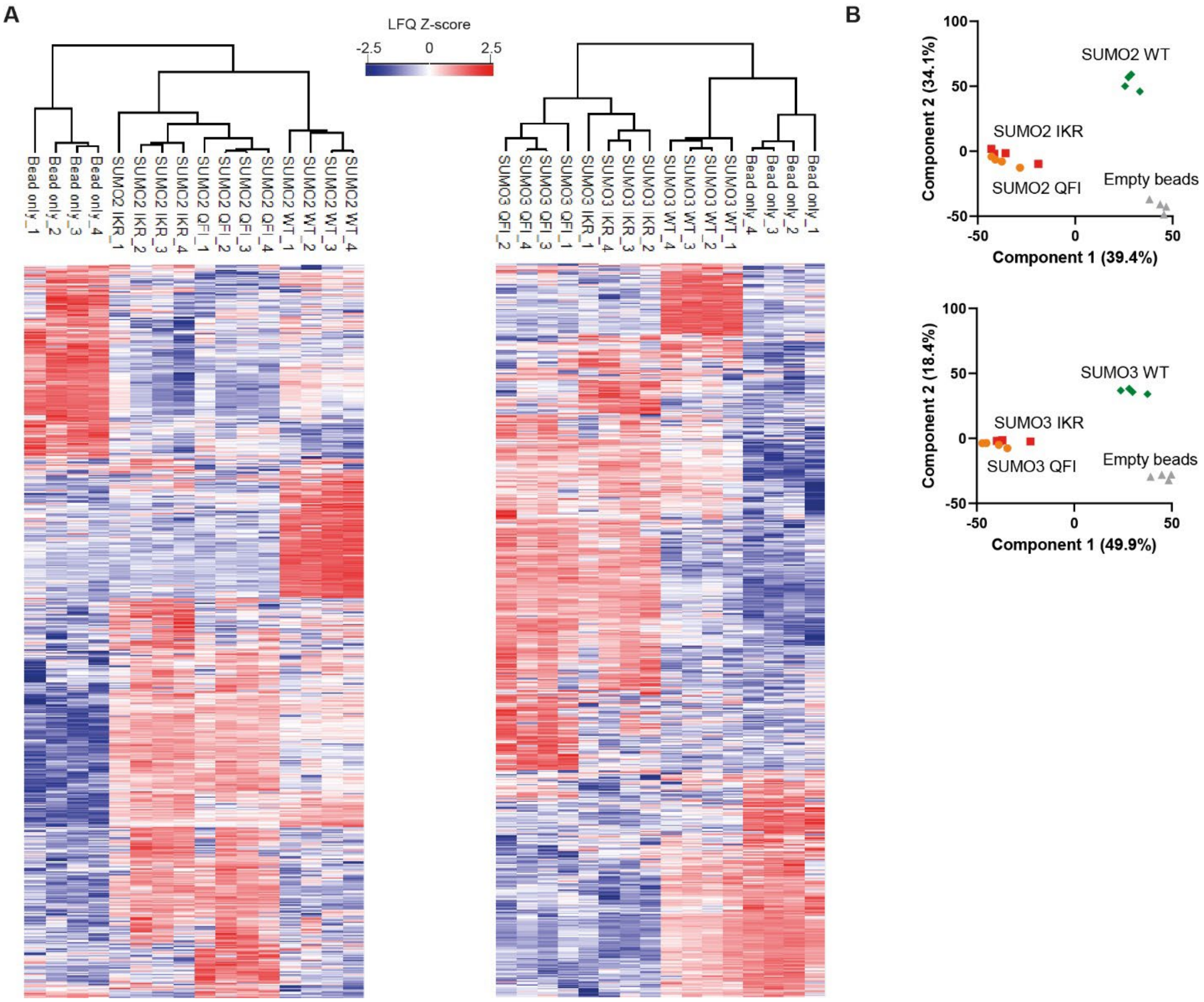

Supplementary Figure 2. Quality control of mass spectrometry data corresponding to Figure 2. (A) Hierarchical clustering by Euclidian distance where each column in the heatmap represents a sample group and each row an identified protein. LFQ intensities were normalized by Z-score. **(B)** Principal component analysis using components with the highest explained variance (component 1 and component 2).

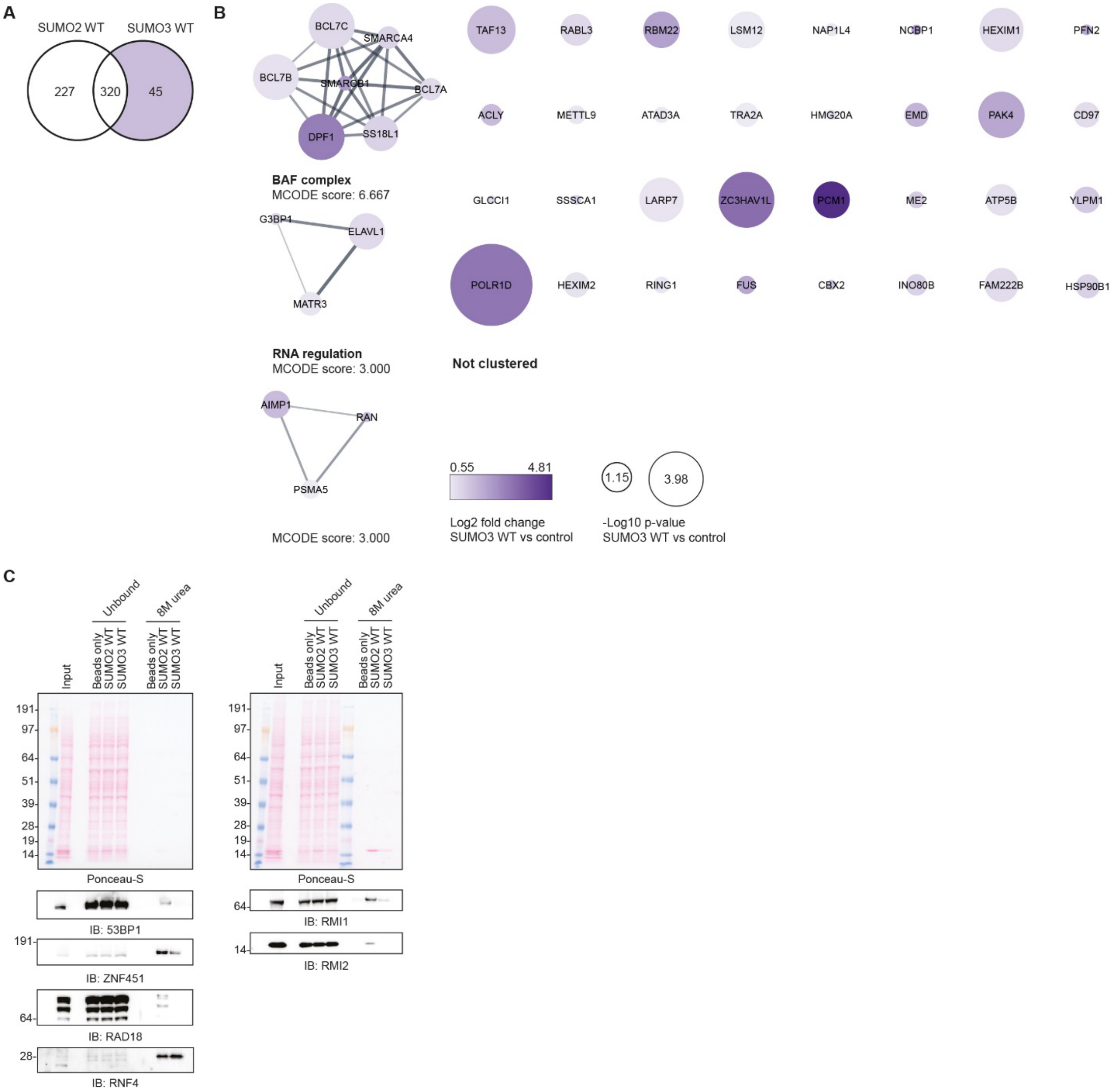

Supplementary Figure 3. Overlap between SUMO2 and SUMO3 interactors corresponding to Figure 2. **(A)** Venn diagram of the identified SUMO2 and SUMO3 interactors in the noncovalent interaction screen. Proteins exclusively binding to His10-SUMO3 were highlighted in purple. **(B)** STRING network of proteins exclusively binding to His10-SUMO3 and not to His10-SUMO2. Node colour and size indicate the log2 fold change and -log p-value, respectively, of the enriched proteins compared to control beads. **(C)** *In vitro* SUMO binding assay as described in Figure 1B comparing His10-SUMO2 and His10-SUMO3 binding. Immunoblotting with antibodies directed against 53BP1, ZNF451, RAD18, RMI1 and RMI18 confirmed preferential or exclusive binding to SUMO2 compared to SUMO3. Anti-RNF4 was used as a positive control for equal binding to both isoforms. n=3 independent experiments.

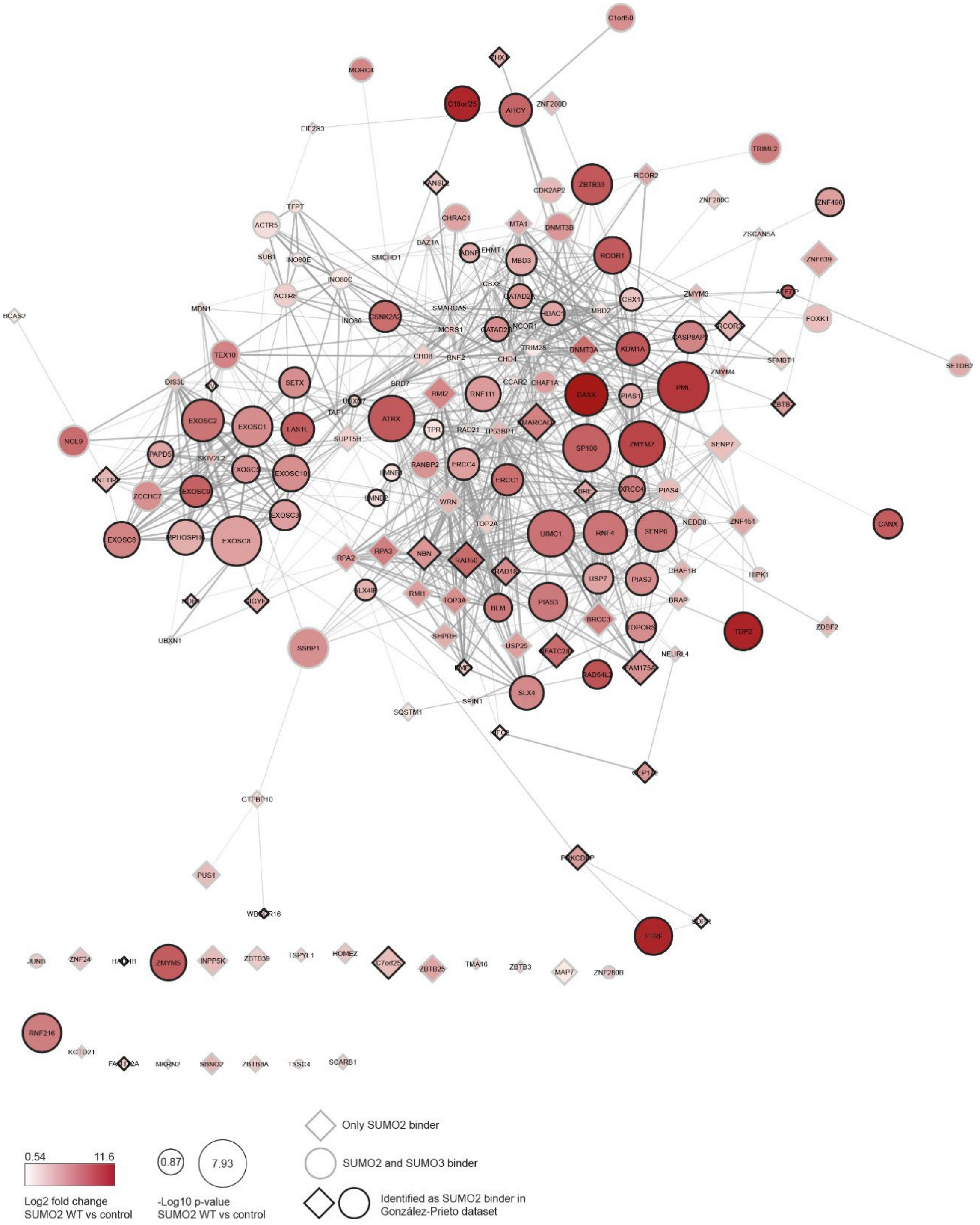

Supplementary Figure 4. STRING network of proteins exclusively binding to SUMO2/3 wildtype and not the SIM-binding groove mutants. STRING network corresponding to Figure 3. Circles represent proteins identified as both SUMO2 and SUMO3 binder; diamonds represent proteins only identified as SUMO2 binder. Black border indicates proteins that we also identified as SUMO2 wildtype binders in a previous interactomics screen. Node colour and size indicate the log2 fold change and -log p-value, respectively, of the enriched proteins compared to the control beads.

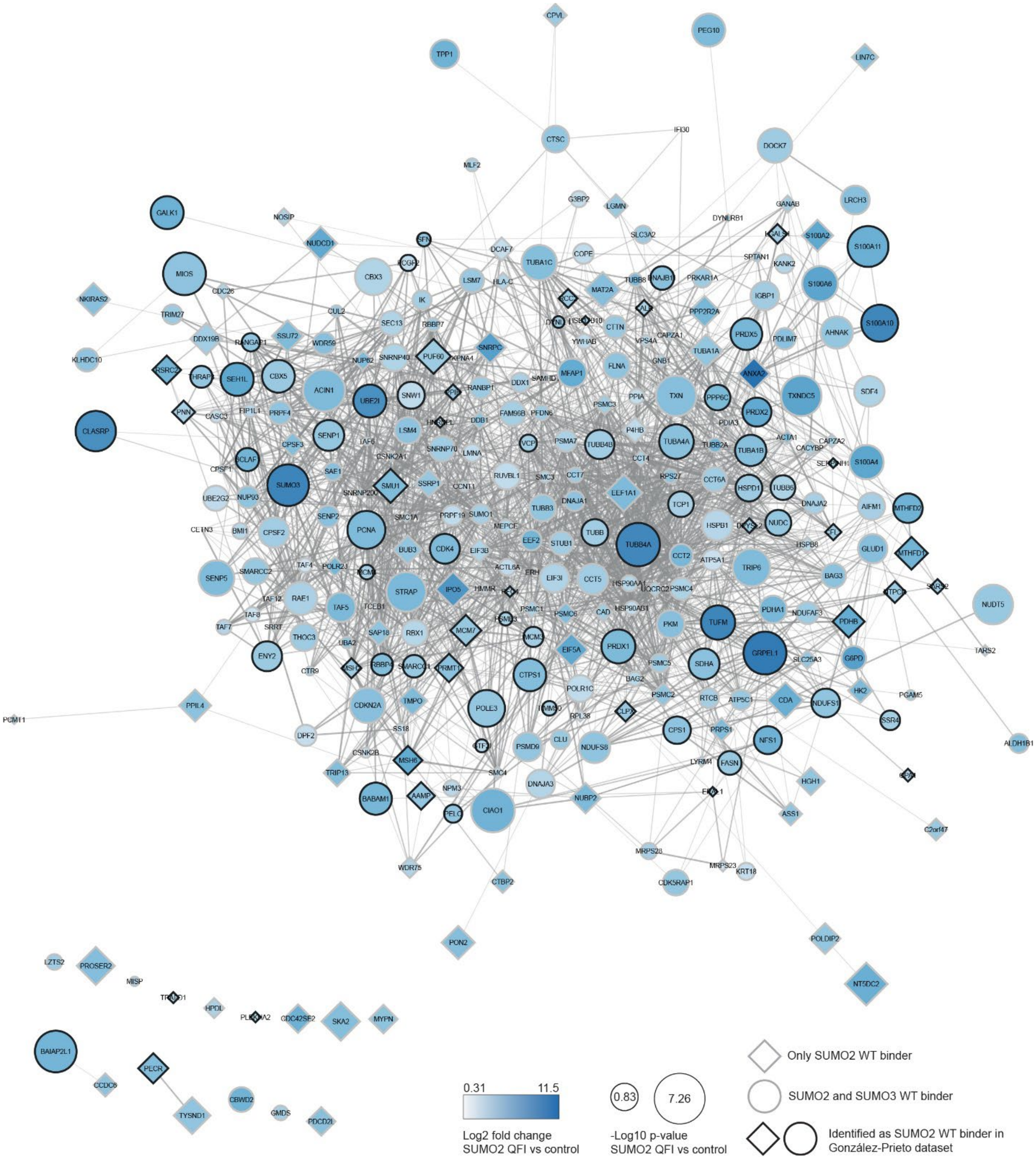

Supplementary Figure 5. STRING network of proteins binding to both SUMO2/3 wildtype and the SIM-binding groove mutants. STRING network corresponding to Figure 4. Circles represent proteins identified as both SUMO2 and SUMO3 binder; diamonds represent proteins only identified as SUMO2 binder. Black border indicates proteins that we also identified as SUMO2 wildtype binders in a previous interactomics screen. Node colour and size indicate the log2 fold change and -log p-value, respectively, of the enriched proteins compared to the control beads.

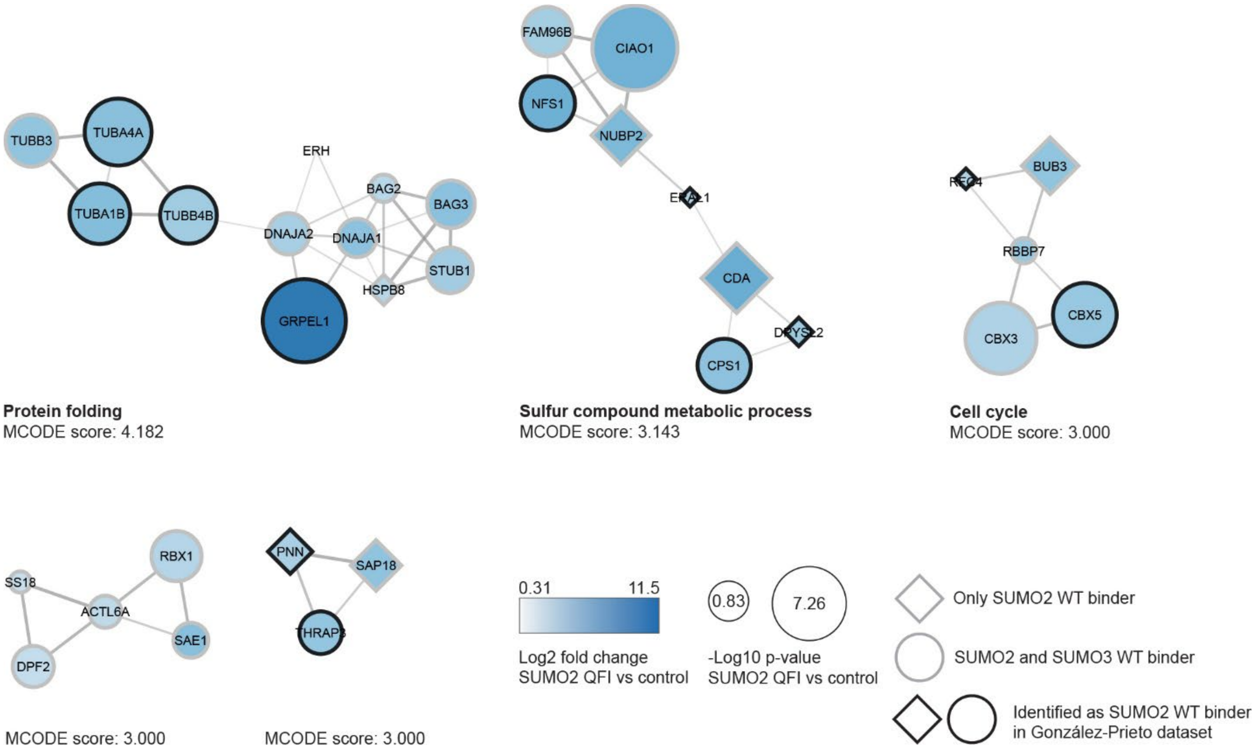

Supplementary Figure 6. MCODE clustering of proteins binding to both SUMO2/3 wildtype and the SIM-binding groove mutants. Remaining MCODE clusters (7-11) corresponding to Figure 4A. Circles represent proteins identified as both SUMO2 and SUMO3 binder; diamonds represent proteins only identified as SUMO2 binder. Black border indicates proteins that we also identified as SUMO2 wildtype binders in a previous interactomics screen. Node colour and size indicate the log2 fold change and -log p-value, respectively, of the enriched proteins compared to the control beads.

A

Preferential SUMO2 WT binders

Protein	SUMO2 WT vs SUMO2 IKR -log p value	SUMO2 WT vs SUMO2 IKR log2 difference	SUMO2 WT vs SUMO2 QFI -log p value	SUMO2 WT vs SUMO2 QFI log2 difference
AAMP	1.2	1.4	2.3	0.5
BABAM1	4.5	2.8	5.5	2.9
CBX3	3.2	1.1	3.5	1.1
CBX5	1.5	0.9	2.0	1.1
CSNK2A1;CSNK2A3	4.9	5.7	4.6	5.3
CSNK2B	5.7	4.5	5.5	4.5
DYNLL1	2.7	3.0	3.3	3.1
GTF2I	1.2	1.1	2.2	1.4
LMNA	3.2	2.8	4.1	3.2
MIOS	2.3	1.0	2.7	1.1
NPM3	2.7	1.6	1.5	1.0
POLE3	2.4	3.2	5.5	2.2
PRPF19	2.3	1.0	1.8	0.8
RANGAP1	3.8	4.5	3.4	3.5
RBBP4	4.7	3.2	4.6	3.1
RBBP7	4.9	1.8	5.9	2.6
RUVBL1	3.7	1.0	2.9	0.7
SAE1	5.1	4.9	4.6	4.5
SENP5	2.3	0.6	4.4	1.7
SUMO1	2.9	1.0	3.9	1.5
UBA2	5.1	6.6	4.5	5.4
VCP	2.6	1.7	2.2	1.6
WDR59	2.0	1.3	2.2	2.1

Preferential SUMO3 WT binders

Protein	SUMO3 WT vs SUMO3 IKR -log p value	SUMO3 WT vs SUMO3 IKR log2 difference	SUMO3 WT vs SUMO3 QFI -log p value	SUMO3 WT vs SUMO3 QFI log2 difference
BMI1	1.8	1.1	1.9	0.9
CSNK2A1;CSNK2A3	3.7	4.1	4.2	4.0
DYNLL1	2.3	2.2	2.8	1.9
PHC1	1.1	1.3	1.9	1.9
RANGAP1	3.4	4.5	3.2	3.0
RBBP4	4.5	1.8	4.9	1.8
RBBP7	3.4	0.6	3.9	0.9
RUVBL1	3.9	1.1	2.7	0.5
SAE1	4.8	4.3	5.8	3.4
VCP	2.0	2.2	2.4	1.6

B

Overlap SIM-independent SUMO2/3 binders with class II SUMO1 binders											
CACYBP	DNAJA1	G6PD	HSP90AA1	NFS1	PRMT1	RTCB	S100A11	TUBB6	UBE2I	VCP
CAD	DNAJA2			NUBP2	PUF60		SMC1A	TUBB8		
CCT4				NUDC			SMC3			
CDK4										
CPSF3										
CUL2										

Supplementary Figure 7. Proteins preferentially binding to SUMO2/3 wildtype compared to the SIM-binding groove mutants. (A) List of proteins preferentially binding to His10-SUMO2 WT compared to IKR and QFI (left), and His10-SUMO3 WT compared to IKR and QFI (right). **(B)** List of proteins that were identified in our screen as common SUMO2/3 WT/QFI/IKR binders and overlap with proteins that were identified in a published interaction screen identifying class II SUMO1 binders.

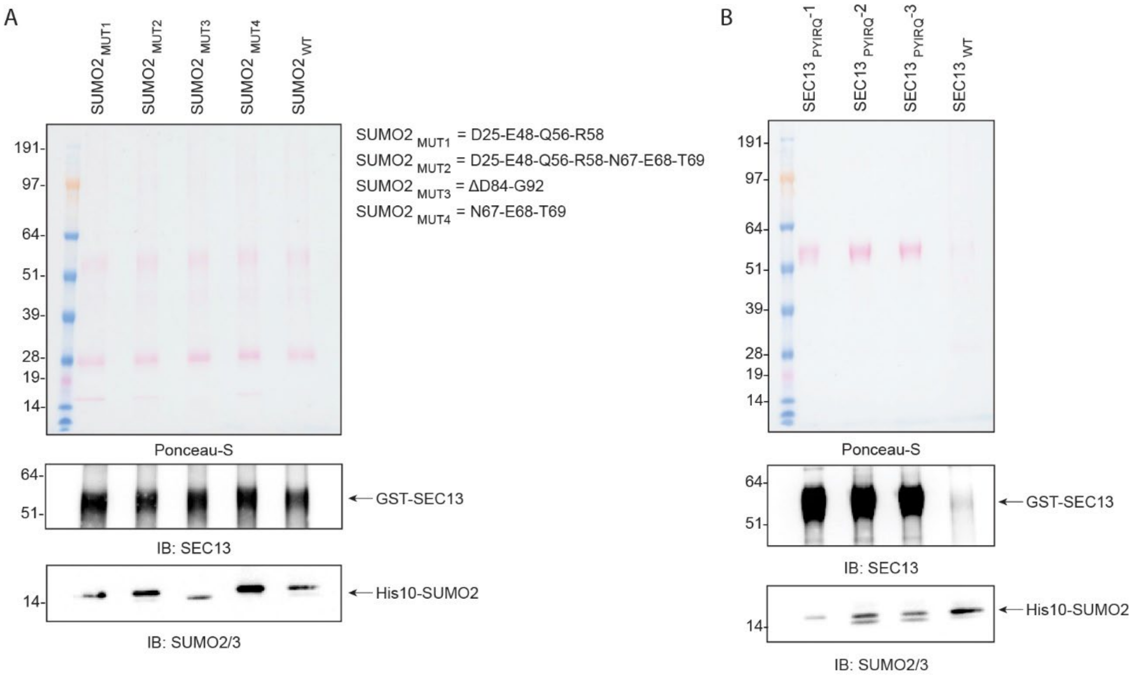

Supplementary Figure 8. Immunoblotting corresponding to Figure 7E-F. (A) Anti-SEC13 and anti-SUMO2/3 immunoblotting of elutions from a GST-pulldown assay with recombinant GST-SEC13 and recombinant His10-SUMO2 wildtype and mutants, corresponding to Figure 7E. n=2 independent experiments. **(B)** Anti-SEC13 and anti-SUMO2/3 immunoblotting of elutions from a GST-pulldown assay with recombinant GST-SEC13 wildtype and P59A-Y79A-I151A-R216A-Q236A mutant and recombinant His10-SUMO2 wildtype, corresponding to Figure 7F. $SEC13_{PYIRQ}$-1, $SEC13_{PYIRQ}$-2, $SEC13_{PYIRQ}$-3 represent elutions from n=3 independent experiments.

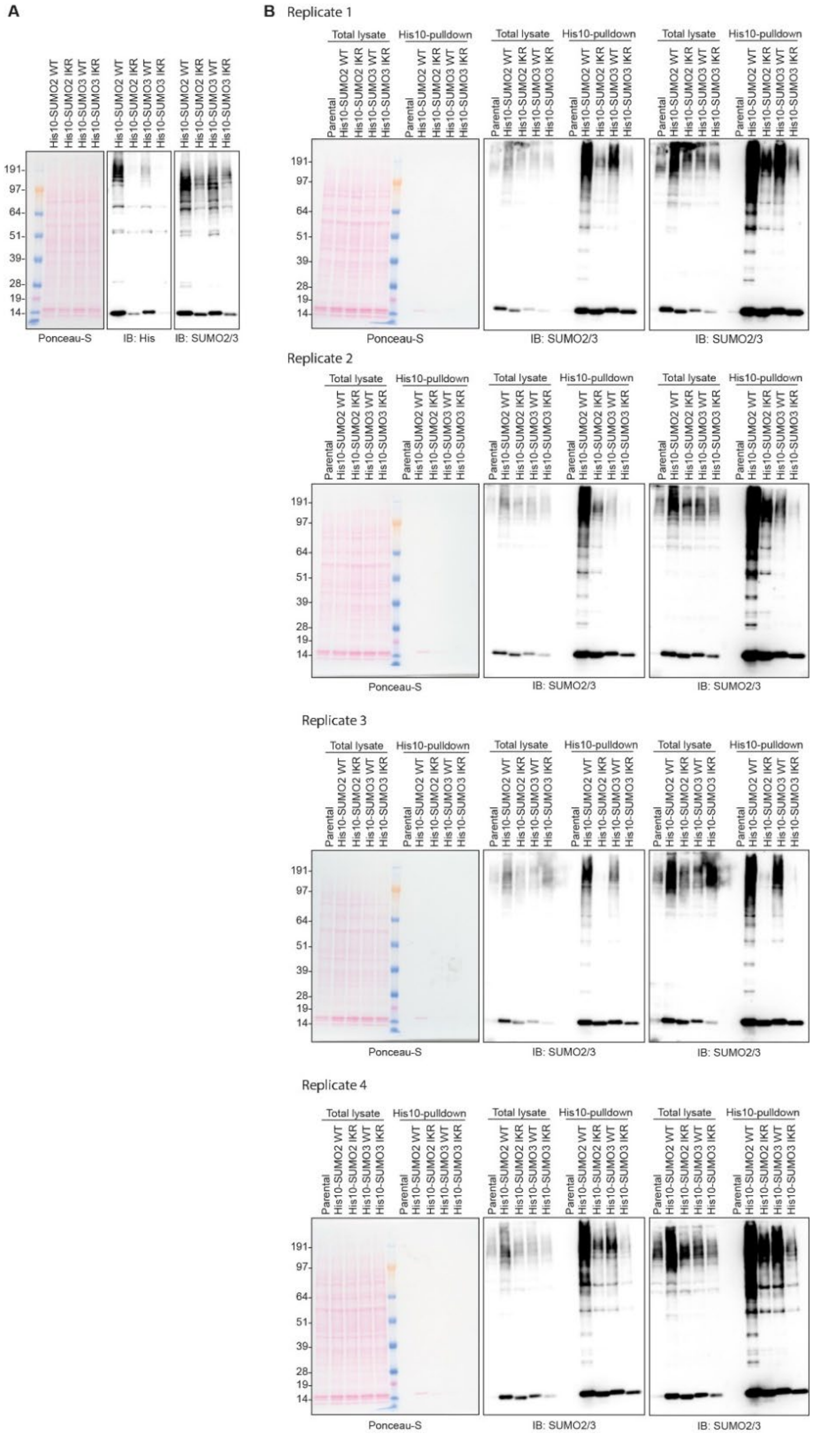

Supplementary Figure 9. Immunoblotting corresponding to Figure 8. (A) Anti-His-tag and anti-SUMO2/3 immunoblotting of HeLa His10-SUMO2/3 WT and IKR cell lines used in the covalent SUMOylation screen. Equal loading of total lysates was verified by Ponceau-S staining. **(B)** Anti-SUMO2/3 immunoblotting of all replicates of the screen, n=4 independent experiments (left: short exposure; right: long exposure). Total lysates and His10-pulldown elutions were analyzed. Equal loading of total lysates was verified by Ponceau-S staining. Replicate 1 in Panel B is duplicated from Main Figure 8 Panel C.

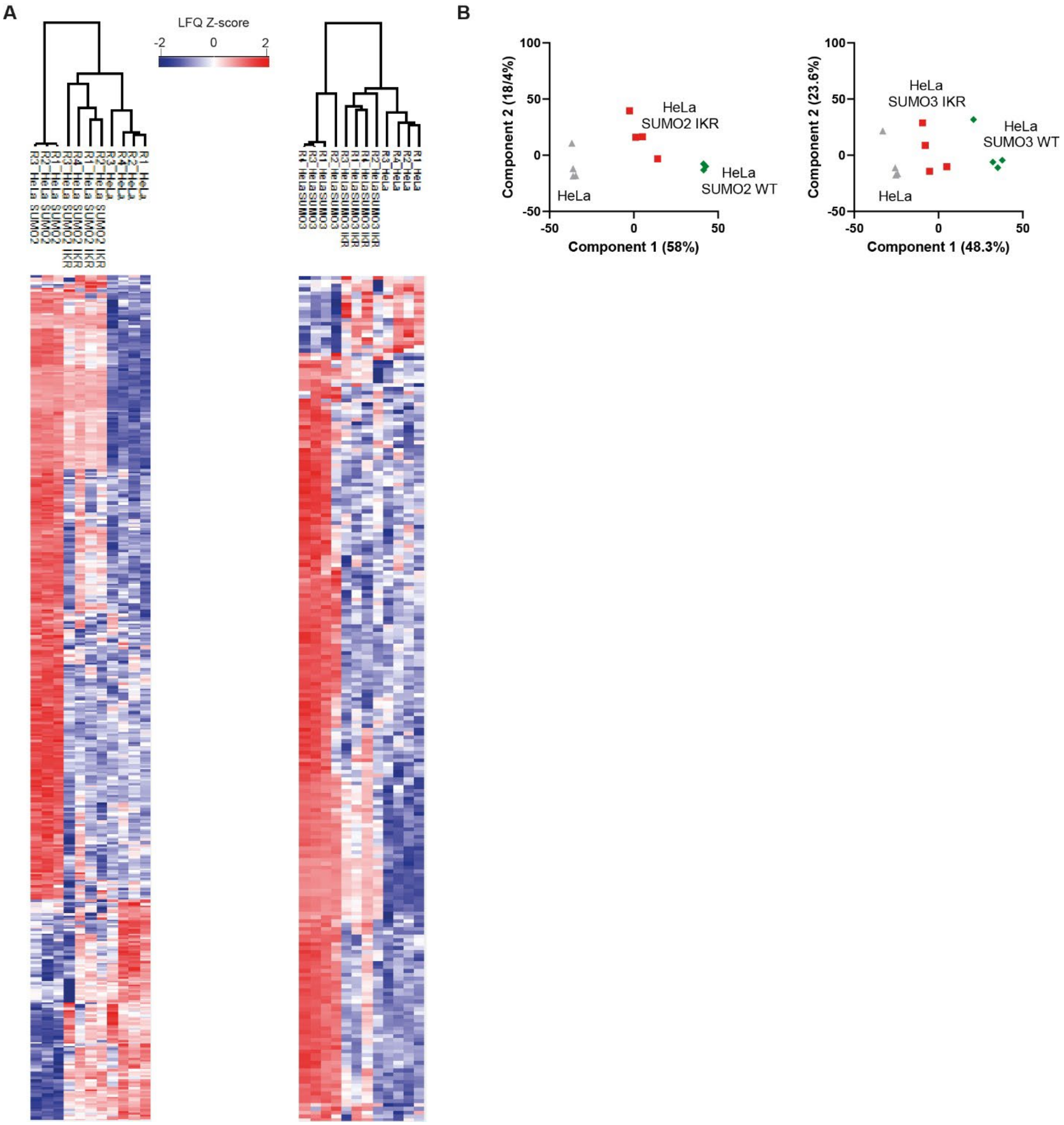

Supplementary Figure 10. Quality control of mass spectrometry data corresponding to Figure 8. (A) Hierarchical clustering by Euclidian distance where each column in the heatmap represents a sample group and each row an identified protein. LFQ intensities were normalized by Z-score. **(B)** Principal component analysis using components with the highest explained variance (component 1 and component 2).

Supplementary Table 5. Key resources

Reagent or resource	Source	Identifier
**Antibodies**		
anti-SUMO2/3 8A2 (IB[a] 1:250)	University of Iowa	8A2
anti-polyHistidine (IB 1:2500)	Sigma-Aldrich	H1029
anti-RNF4 (IB 1:5000)	Eurogentec	*(46)*
anti-SEC13 (IB 1:1000)	Proteintech	15391-1-AP
anti-SEH1L (IB 1:1000)	Abcam	ab218531
anti-TAF5 (IB 1:1000)	Bethyl	A303-686A
anti-53BP1 (IB 1:1000)	Bethyl	A300-272A
anti-ZNF451 (IB 1:1000)	Andrea Pichler	*(47)*
anti-RMI1 (IB 1:1000)	Proteintech	14630-1-AP
anti-RMI2 (IB 1:1000)	Abcam	ab122685
anti-RAD18 (IB 1:1000)	Bethyl	A301-340A
**Oligonucleotides**		
SUMO2 IKR FWD: 5'-GCTCCGTGGTGCAGTTCAAGgcCgcGgcGCACACGCCGCTGAGCAAGCT-3'	This study	N/A
SUMO2 IKR REV: 5'-AGCTTGCTCAGCGGCGTGTGCgcCgcGgcCTTGAACTGCACCACGGAGC-3'	This study	N/A
SUMO2 QFI FWD: 5'-GGCAGGACGGCTCCGTGGTGGCGGCCAAGGCCAAGAGGCACACGCCGCTGAG-3'	This study	N/A
SUMO2 QFI REV: 5'-CTCAGCGGCGTGTGCCTCTTGGCCTTGGCCGCCACCACGGAGCCGTCCTGCC-3'	This study	N/A
SUMO3 IKR FWD: 5'-GTTCTGTGGTGCAGTTTAAGgcTgcGgcGCATACACCACTTAGTAAACT-3'	This study	N/A
SUMO3 IKR REV: 5'-AGTTTACTAAGTGGTGTATGCgcCgcAgcCTTAAACTGCACCACAGAAC-3'	This study	N/A
SUMO3 QFI FWD: 5'-GGCAGGATGGTTCTGTGGTGGCGGCTAAGGCTAAGAGGCATACACCACTTAG-3'	This study	N/A
SUMO3 QFI REV: 5'-CTAAGTGGTGTATGCCTCTTAGCCTTAGCCGCCACCACAGAACCATCCTGCC-3'	This study	N/A
SEC13 STOP FW: 5'-GGCCAGCAGAACGAGCAGTGATGCCCAACTTTCTTGTAC-3'	This study	N/A
SEC13 STOP REV: 5'-GTACAAGAAAGTTGGGCATCACTGCTCGTTCTGCTGGCC-3'	This study	N/A
SEH1L BP FW: 5'-GGGGACAAGTTTGTACAAAAAAGCAGGCTTCatgtttgtggctcgcagcatcg-3'	This study	N/A

SEH1L BP REV: 5'-GGGGACCACTTTGTACAAGAAAGCTGGGTCtcagctgtgctttctgccagcag-3'	This study	N/A
**Recombinant DNA**		
pUC57-His10-SUMO2 WT	GenScript	N/A
pUC57-His10-SUMO3 WT	GenScript	N/A
pUC57-His10-SUMO2 D25A-E48A-Q56A-R58A	GenScript	N/A
pUC57-His10-SUMO2 N67A-E68A-T69A	GenScript	N/A
pUC57-His10-SUMO2 D25A-E48A-Q56A-R58A-N67A-E68A-T69A	GenScript	N/A
pUC57-His10-SUMO2 ΔD84-G92	GenScript	N/A
pUC57-His10-SUMO2 IKR	This study	N/A
pUC57-His10-SUMO3 IKR	This study	N/A
pUC57-His10-SUMO2 QFI	This study	N/A
pUC57-His10-SUMO3 QFI	This study	N/A
pET11a-His10-SUMO2 WT	This study	N/A
pET11a-His10-SUMO3 WT	This study	N/A
pET11a-His10-SUMO2 IKR	This study	N/A
pET11a-His10-SUMO3 IKR	This study	N/A
pET11a-His10-SUMO2 QFI	This study	N/A
pET11a-His10-SUMO3 QFI	This study	N/A
pET11a-His10-SUMO2 D25A-E48A-Q56A-R58A	This study	N/A
pET11a-His10-SUMO2 N67A-E68A-T69A	This study	N/A
pET11a-His10-SUMO2 D25A-E48A-Q56A-R58A-N67A-E68A-T69A	This study	N/A
pET11a-His10-SUMO2 ΔD84-G92	This study	N/A
pLV-IRES-puro-His10-SUMO2 WT	This study	N/A
pLV-IRES-puro-His10-SUMO3 WT	This study	N/A
pLV-IRES-puro-His10-SUMO2 IKR	This study	N/A
pLV-IRES-puro-His10-SUMO3 IKR	This study	N/A
pRK5-SEH1L cDNA	Addgene	154216
pDONR223-SEC13 ORF	Sigma-Aldrich MISSION® TRC3 Human ORF Collection	Legacy clone name: ORF005306.1_s300c1
pDEST15-SEH1L	This study	N/A
pDEST15-SEC13	This study	N/A
pDONR207-SEC13 P59A-Y79A-I151A-R216A-Q236A	GenScript	N/A
pDEST15-SEC13 P59A-Y79A-I151A-R216A-Q236A	GenScript	N/A

[a]IB: immunoblotting

Supplementary Table 1. Mass spectrometry results for noncovalent SUMO2 wildtype and SIM-binding groove mutant binders.

Supplementary Table 2. Mass spectrometry results for noncovalent SUMO3 wildtype and SIM-binding groove mutant binders.

Supplementary Table 3. Mass spectrometry results for covalent SUMO targets of SUMO2 wildtype and SIM-binding groove mutants.

Supplementary Table 4. Mass spectrometry results for covalent SUMO targets of SUMO3 wildtype and SIM-binding groove mutants.

Supplementary Movie 1. AlphaFold 3 modelling of SUMO2-SEC13 interaction. AlphaFold 3 modelling of SUMO2-SEC13 interaction corresponding to Figure 7D. Key residues involved in the interaction are highlighted.

REFERENCES AND NOTES

1. A. C. O. Vertegaal, Signalling mechanisms and cellular functions of SUMO. *Nat. Rev. Mol. Cell Biol.* **23**, 715–731 (2022).

2. J. Keiten-Schmitz, K. Schunck, S. Müller, SUMO chains rule on chromatin occupancy. *Front. Cell Dev. Biol.* **7**, 343 (2019).

3. N. S. Jansen, A. C. O. Vertegaal, A chain of events: Regulating target proteins by SUMO polymers. *Trends Biochem. Sci.* **46**, 113–123 (2021).

4. C. M. Hickey, N. R. Wilson, M. Hochstrasser, Function and regulation of SUMO proteases. *Nat. Rev. Mol. Cell Biol.* **13**, 755–766 (2012).

5. A. Pichler, C. Fatouros, H. Lee, N. Eisenhardt, SUMO conjugation - A mechanistic view. *Biomol. Concepts* **8**, 13–36 (2017).

6. J. Lascorz, J. Codina-Fabra, D. Reverter, J. Torres-Rosell, SUMO-SIM interactions: From structure to biological functions. *Semin. Cell Dev. Biol.* **132**, 193–202 (2022).

7. T. Y. Yau, W. Sander, C. Eidson, A. J. Courey, SUMO interacting motifs: Structure and function. *Cells* **10**, (2021).

8. J. Song, L. K. Durrin, T. A. Wilkinson, T. G. Krontiris, Y. Chen, Identification of a SUMO-binding motif that recognizes SUMO-modified proteins. *Proc. Natl. Acad. Sci. U.S.A.* **101**, 14373–14378 (2004).

9. R. Ullmann, C. D. Chien, M. L. Avantaggiati, S. Muller, An acetylation switch regulates SUMO-dependent protein interaction networks. *Mol. Cell* **46**, 759–770 (2012).

10. C.-C. Chang, M. T. Naik, Y.-S. Huang, J.-C. Jeng, P.-H. Liao, H.-Y. Kuo, C.-C. Ho, Y.-L. Hsieh, C.-H. Lin, N.-J. Huang, N. M. Naik, Camy C-H Kung, S.-Y. Lin, R.-H. Chen, K.-S. Chang, T.-H. Huang, H.-M. Shih, Structural and functional roles of Daxx SIM phosphorylation in SUMO paralog-selective binding and apoptosis modulation. *Mol. Cell* **42**, 62–74 (2011).

11. Anamika, L. Spyracopoulos, Molecular basis for phosphorylation-dependent SUMO recognition by the DNA repair protein RAP80. *J. Biol. Chem.* **291**, 4417–4428 (2016).

12. X. H. Mascle, M. Lussier-Price, L. Cappadocia, P. Estephan, L. Raiola, J. G. Omichinski, M. Aubry, Identification of a non-covalent ternary complex formed by PIAS1, SUMO1, and UBC9 proteins involved in transcriptional regulation. *J. Biol. Chem.* **288**, 36312–36327 (2013).

13. P. Stehmeier, S. Muller, Phospho-regulated SUMO interaction modules connect the SUMO system to CK2 signaling. *Mol. Cell* **33**, 400–409 (2009).

14. C. M. Hecker, M. Rabiller, K. Haglund, P. Bayer, I. Dikic, Specification of SUMO1- and SUMO2-interacting motifs. *J. Biol. Chem.* **281**, 16117–16127 (2006).

15. A. M. Sriramachandran, K. Meyer-Teschendorf, S. Pabst, H. D. Ulrich, N. H. Gehring, K. Hofmann, G. J. K. Praefcke, R. J. Dohmen, Arkadia/RNF111 is a SUMO-targeted ubiquitin ligase with preference for substrates marked with SUMO1-capped SUMO2/3 chain. *Nat. Commun.* **10**, 3678 (2019).

16. E. Pilla, U. Möller, G. Sauer, F. Mattiroli, F. Melchior, R. Geiss-Friedlander, A novel SUMO1-specific interacting motif in dipeptidyl peptidase 9 (DPP9) that is important for enzymatic regulation. *J. Biol. Chem.* **287**, 44320–44329 (2012).

17. A. D. Capili, C. D. Lima, Structure and analysis of a complex between SUMO and Ubc9 illustrates features of a conserved E2-Ubl interaction. *J. Mol. Biol.* **369**, 608–618 (2007).

18. P. Knipscheer, W. J. van Dijk, J. V. Olsen, M. Mann, T. K. Sixma, Noncovalent interaction between Ubc9 and SUMO promotes SUMO chain formation. *EMBO J.* **26**, 2797–2807 (2007).

19. K. Brüninghoff, S. Wulff, W. Dörner, R. Geiss-Friedlander, H. D. Mootz, A photo-crosslinking approach to identify class II SUMO-1 binders. *Front. Chem.* **10**, 900989 (2022).

20. J. R. Danielsen, L. K. Povlsen, B. H. Villumsen, W. Streicher, J. Nilsson, M. Wikström, S. Bekker-Jensen, N. Mailand, DNA damage-inducible SUMOylation of HERC2 promotes RNF8 binding via a novel SUMO-binding Zinc finger. *J. Cell Biol.* **197**, 179–187 (2012).

21. C. Diehl, M. Akke, S. Bekker-Jensen, N. Mailand, W. Streicher, M. Wikström, Structural analysis of a complex between small ubiquitin-like modifier 1 (SUMO1) and the ZZ domain of CREB-binding protein (CBP/p300) reveals a new interaction surface on SUMO. *J. Biol. Chem.* **291**, 12658–12672 (2016).

22. M. J. Cabello-Lobato, M. Jenner, M. Cisneros-Aguirre, K. Brüninghoff, Z. Sandy, Isabelle C. da Costa, Thomas A. Jowitt, Christian M. Loch, Stephen P. Jackson, Q. Wu, Henning D. Mootz, Jeremy M. Stark, Matthew J. Cliff, Christine K. Schmidt, Microarray screening reveals two non-conventional SUMO-binding modules linked to DNA repair by non-homologous end-joining. *Nucleic Acids Res.* **50**, 4732–4754 (2022).

23. E. Cox, W. Hwang, I. Uzoma, J. Hu, C. M. Guzzo, J. Jeong, M. J. Matunis, J. Qian, H. Zhu, S. Blackshaw, Global analysis of SUMO-binding proteins identifies SUMOylation as a key regulator of the INO80 chromatin remodeling complex. *Mol. Cell. Proteomics* **16**, 812–823 (2017).

24. E. Aguilar-Martinez, X. Chen, A. Webber, A. P. Mould, A. Seifert, R. T. Hay, A. D. Sharrocks, Screen for multi-SUMO-binding proteins reveals a multi-SIM-binding mechanism for recruitment of the transcriptional regulator ZMYM2 to chromatin. *Proc. Natl. Acad. Sci. U.S.A.* **112**, E4854–E4863 (2015).

25. R. González-Prieto, K. Eifler-Olivi, L. A. Claessens, E. Willemstein, Z. Xiao, Cami M P Talavera Ormeno, H. Ovaa, H. D. Ulrich, A. C. O. Vertegaal, Global non-covalent SUMO interaction networks reveal SUMO-dependent stabilization of the non-homologous end joining complex. *Cell Rep.* **34**, 108691 (2021).

26. K. Brüninghoff, A. Aust, K. F. Taupitz, S. Wulff, W. Dörner, H. D. Mootz, Identification of SUMO binding proteins enriched after covalent photo-cross-linking. *ACS Chem. Biol.* **15**, 2406–2414 (2020).

27. C. M. Guzzo, A. Ringel, E. Cox, I. Uzoma, H. Zhu, S. Blackshaw, C. Wolberger, M. J. Matunis, Characterization of the SUMO-binding activity of the myeloproliferative and mental retardation (MYM)-type zinc fingers in ZNF261 and ZNF198. *PLOS ONE* **9**, e105271 (2014).

28. J. Zhu, S. Zhu, C. M. Guzzo, N. A. Ellis, K. S. Sung, C. Y. Choi, M. J. Matunis, Small ubiquitin-related modifier (SUMO) binding determines substrate recognition and paralog-selective SUMO modification. *J. Biol. Chem.* **283**, 29405–29415 (2008).

29. R. González-Prieto, S. A. Cuijpers, M. S. Luijsterburg, H. van Attikum, A. C. Vertegaal, SUMOylation and PARylation cooperate to recruit and stabilize SLX4 at DNA damage sites. *EMBO Rep.* **16**, 512–519 (2015).

30. L. A. Claessens, M. Verlaan-de Vries, I. J. de Graaf, A. C. O. Vertegaal, SENP6 regulates localization and nuclear condensation of DNA damage response proteins by group deSUMOylation. *Nat. Commun.* **14**, 5893 (2023).

31. N. Pashkova, L. Gakhar, S. C. Winistorfer, L. Yu, S. Ramaswamy, R. C. Piper, WD40 repeat propellers define a ubiquitin-binding domain that regulates turnover of F box proteins. *Mol. Cell* **40**, 433–443 (2010).

32. J. Abramson, J. Adler, J. Dunger, R. Evans, T. Green, A. Pritzel, O. Ronneberger, L. Willmore, A. J. Ballard, J. Bambrick, S. W. Bodenstein, D. A. Evans, C. C. Hung, M. O'Neill, D. Reiman, K. Tunyasuvunakool, Z. Wu, A. Žemgulytė, E. Arvaniti, C. Beattie, O. Bertolli, A. Bridgland, A. Cherepanov, M. Congreve, A. I. Cowen-Rivers, A. Cowie, M. Figurnov, F. B. Fuchs, H. Gladman, R. Jain, Y. A. Khan, C. M. R. Low, K. Perlin, A. Potapenko, P. Savy, S. Singh, A. Stecula, A. Thillaisundaram, C. Tong, S. Yakneen, E. D. Zhong, M. Zielinski, A. Žídek, V. Bapst, P. Kohli, M. Jaderberg, D. Hassabis, J. M. Jumper, Accurate structure prediction of biomolecular interactions with AlphaFold 3. *Nature* **630**, 493–500 (2024).

33. Y. Wang, X.-J. Hu, X.-D. Zou, X.-H. Wu, Z.-Q. Ye, Y.-D. Wu, WDSPdb: A database for WD40-repeat proteins. *Nucleic Acids Res.* **43**, D339–D344 (2014).

34. E. Meulmeester, M. Kunze, H. H. Hsiao, H. Urlaub, F. Melchior, Mechanism and consequences for paralog-specific sumoylation of ubiquitin-specific protease 25. *Mol. Cell* **30**, 610–619 (2008).

35. D. Y. Lin, Y. S. Huang, J. C. Jeng, H. Y. Kuo, C. C. Chang, T. T. Chao, C. C. Ho, Y. C. Chen, T. P. Lin, H. I. Fang, C. C. Hung, C. S. Suen, M. J. Hwang, K. S. Chang, G. G. Maul, H. M.

Shih, Role of SUMO-interacting motif in Daxx SUMO modification, subnuclear localization, and repression of sumoylated transcription factors. *Mol. Cell* **24**, 341–354 (2006).

36. T. Moriuchi, F. Hirose, SUMOylation of RepoMan during late telophase regulates dephosphorylation of lamin A. *J. Cell Sci.* **134**, jcs247171 (2021).

37. U. Sahin, O. Ferhi, M. Jeanne, S. Benhenda, C. Berthier, F. Jollivet, M. Niwa-Kawakita, O. Faklaris, N. Setterblad, H. de Thé, V. Lallemand-Breitenbach, Oxidative stress-induced assembly of PML nuclear bodies controls sumoylation of partner proteins. *J. Cell Biol.* **204**, 931–945 (2014).

38. M. H. Tatham, M. C. Geoffroy, L. Shen, A. Plechanovova, N. Hattersley, E. G. Jaffray, J. J. Palvimo, R. T. Hay, RNF4 is a poly-SUMO-specific E3 ubiquitin ligase required for arsenic-induced PML degradation. *Nat. Cell Biol.* **10**, 538–546 (2008).

39. S. L. Poulsen, R. K. Hansen, S. A. Wagner, L. van Cuijk, G. J. van Belle, W. Streicher, M. Wikström, C. Choudhary, A. B. Houtsmuller, J. A. Marteijn, S. Bekker-Jensen, N. Mailand, RNF111/Arkadia is a SUMO-targeted ubiquitin ligase that facilitates the DNA damage response. *J. Cell Biol.* **201**, 797–807 (2013).

40. K. Wagner, K. Kunz, T. Piller, G. Tascher, S. Hölper, P. Stehmeier, J. Keiten-Schmitz, M. Schick, U. Keller, S. Müller, The SUMO Isopeptidase SENP6 Functions as a rheostat of chromatin residency in genome maintenance and chromosome dynamics. *Cell Rep.* **29**, 480–494.e5 (2019).

41. L. Cappadocia, A. Pichler, C. D. Lima, Structural basis for catalytic activation by the human ZNF451 SUMO E3 ligase. *Nat. Struct. Mol. Biol.* **22**, 968–975 (2015).

42. K. Kaur, H. Park, N. Pandey, Y. Azuma, R. N. De Guzman, Identification of a new small ubiquitin-like modifier (SUMO)-interacting motif in the E3 ligase PIASy. *J. Biol. Chem.* **292**, 10230–10238 (2017).

43. A. Werner, A. Flotho, F. Melchior, The RanBP2/RanGAP1*SUMO1/Ubc9 complex is a multisubunit SUMO E3 ligase. *Mol. Cell* **46**, 287–298 (2012).

44. I. A. Hendriks, A. C. Vertegaal, Label-free identification and quantification of SUMO target proteins. *Methods Mol. Biol.* **1475**, 171–193 (2016).

45. J. Rappsilber, M. Mann, Y. Ishihama, Protocol for micro-purification, enrichment, pre-fractionation and storage of peptides for proteomics using StageTips. *Nat. Protoc.* **2**, 1896–1906 (2007).

46. R. Vyas, R. Kumar, F. Clermont, A. Helfricht, P. Kalev, P. Sotiropoulou, I. A. Hendriks, E. Radaelli, T. Hochepied, C. Blanpain, A. Sablina, H. van Attikum, J. V. Olsen, A. G. Jochemsen, A. C. O. Vertegaal, J.-C. Marine, RNF4 is required for DNA double-strand break repair in vivo. *Cell Death Differ.* **20**, 490–502 (2013).

47. N. Eisenhardt, V. K. Chaugule, S. Koidl, M. Droescher, E. Dogan, J. Rettich, P. Sutinen, S. Y. Imanishi, K. Hofmann, J. J. Palvimo, A. Pichler, A new vertebrate SUMO enzyme family reveals insights into SUMO-chain assembly. *Nat. Struct. Mol. Biol.* **22**, 959–967 (2015).
